# Exploratory study of the acute and mid-term effects of using a novel dynamic meeting environment (Aeris^®^) on cognitive performance and neurophysiological responses

**DOI:** 10.3389/fnhum.2023.1282728

**Published:** 2023-11-23

**Authors:** Achraf Ammar, Mohamed Ali Boujelbane, Marvin Leonard Simak, Irene Fraile-Fuente, Khaled Trabelsi, Bassem Bouaziz, Nikolas Rizzi, Wolfgang I. Schöllhorn

**Affiliations:** ^1^Department of Training and Movement Science, Institute of Sport Science, Johannes Gutenberg-University Mainz, Mainz, Germany; ^2^Interdisciplinary Laboratory in Neurosciences, Physiology and Psychology: Physical Activity, Health and Learning (LINP2), UFR STAPS (Faculty of Sport Sciences), UPL, Paris Nanterre University, Nanterre, France; ^3^High Institute of Sport and Physical Education of Sfax, University of Sfax, Sfax, Tunisia; ^4^Research Unit: Physical Activity, Sport, and Health, UR18JS01, National Observatory of Sport, Tunis, Tunisia; ^5^Research Laboratory, Education, Motricity, Sport and Health (EM2S), LR15JS01, High Institute of Sport and Physical Education of Sfax, University of Sfax, Sfax, Tunisia; ^6^MIRACL Laboratory, Higher Institute of Computer Science and Multimedia of Sfax, University of Sfax, Sfax, Tunisia

**Keywords:** mental workload, EEG, HRV, cognitive performance, attention, vigilance, collaborative tasks, brain activity

## Abstract

The purpose of the present study was to assess the acute and mid-term effects of the dynamic aeris^®^-meeting- environment on brain activity, cognitive performance, heart rate variability (HRV), sleepiness, mental workload (EEG-MWI), as well as local experienced discomfort (LED) in healthy adults. Twenty-four healthy adults (16 females, age: 25.2 ± 3.1 years old) were randomly assigned to either the control (i.e., conventional meeting environment, CG) or experimental (Aeris^®^ dynamic meeting-environment, DG) group with a 1:1 allocation. Participants reported to the laboratory on two test sessions separated by a 2-week intervention period (5 meetings of 90 min each week). Spontaneous resting EEG and HRV activities, as well as attentional (D2-R test) and vigilance (PVT) cognitive performances, sleepiness perceptions, and EEG-MWI, were recorded at the beginning of each test session and immediately following the 90-min meeting. The LED was measured pre- and post-intervention. The changes (Δ) from pre- to post-90 min meeting and from pre- to post- intervention were computed to further examine the acute and mid-term effects, respectively. Compared to the CG, the DG showed higher Δ (pre-post 90 min-meeting) in fronto-central beta (*z* = −2.41, *p* = 0.016, *d* = 1.10) and gamma (*z* = −2.34, *p* = 0.019, *d* = 0.94) frequencies at post-intervention. From pre- to post-intervention, only the DG group showed a significant increase in fronto-central gamma response (Δ) to the meeting session (*z* = −2.09, *p* = 0.04, *d* = 1.08). The acute use of the Aeris^®^-meeting-environment during the 90-min meeting session seems to be supportive for (i) maintaining vigilance performance, as evidenced by the significant increase in N-lapses from pre- to post-90 min session only in the CG (*p* = 0.04, *d* = 0.99, Δ = 2.5 ± 3 lapses), and (ii) improving alertness, as evidenced by the lower sleepiness score (*p* = 0.05, *d* = −0.84) in DG compared to CG. The mid-term use of such an environment showed to blind the higher baseline values of EEG-MWI recorded in DG compared to CG (*p* = 0.01, *d* = 1.05) and may prevent lower-back discomfort (i.e., a significant increase only in CG with *p* = 0.05 and *d* = 0.78), suggesting a less mentally and physically exhausting meeting in this environment. There were no acute and/or mid-term effects of the dynamic meeting environment on any of the HRV parameters. These findings are of relevance in the field of neuroergonomics, as they give preliminary support to the advantages of meeting in a dynamic office compared to a static office environment.

## 1 Introduction

Contemporary scientific investigations have consistently revealed the adverse impact of sedentariness and the favorable impact of exercise on physical and cognitive performance as well as on mental wellbeing (Chang et al., 2012; Rebar et al., 2015; World Health Organization, 2018). In addition to the short-term impact of gross motor movements on brain activity, there is a growing interest in investigating the medium-term effects of exercise interventions on learning abilities, encompassing both motor and cognitive domains (Bidzan-Bluma and Lipowska, 2018). In classrooms and working offices, where students and office workers spend > 70% of their study/work time sitting (Cardon et al., 2004; Slomski, 2022), physical activity can be increased by changing the traditional environment into one that encourages regular movement and active behaviors (Rice and Howell, 2000). In this context, dynamic office furniture is increasingly used in office environments to help reduce the negative effects of sedentary work on workers’ psychological and physical health. However, there is still limited systematic investigation into the impact of movement-promoting and sensory-stimulating office environments (Henz and Schöllhorn, 2019). Indeed, only a few recent studies emphasized the effects of dynamic office furniture on various psychophysiological parameters in both office workers and students and showed conflicting results (Chambers et al., 2019). Horswill et al. (2017) showed a significantly higher score in executive function, measured using the Stroop Work Color Test, while engaging in standing workstations compared to sitting workstations. In the same way, Radwan et al. (2022) investigated the effects of using a dynamic office chair on cognitive performance among office workers. The results showed that by using this dynamic chair, workers improved their cognitive performance, specifically their working memory. Meanwhile, Smith et al. (2022) examined the effects of using a sit-stand desk on upper-limb musculoskeletal disorders among office workers and showed a reduced risk of upper-limb musculoskeletal disorders in this environment. On the other hand, no significant differences in working memory, processing speed, or attention were reported between sitting- and standing-based environments at a week’s interval in office workers (Russell et al., 2016). Similarly, Schwartz et al. (2019) investigated the effect of 23 weeks of using a standing vs. seated-based environment on cognitive abilities and demonstrated the absence of any measurable difference in working speed or attention. More surprisingly, a more recent study by Kang et al. (2021) showed the use of the standing workstation degraded attention and executive function. This discrepancy between the results has been highlighted in a recent review article by Chambers et al. (2019), who failed to reach a conclusion on cognitive abilities under sitting and standing conditions.

In addition to the studies on office workers, there have also been studies examining the short-term (Ee et al., 2018; Tanaka and Noi, 2022), mid-term (Ayala et al., 2016; Wick et al., 2018; Kidokoro et al., 2019; Rhee and Benden, 2020), and long-term (Parry et al., 2019) effects of dynamic office furniture on students’ executive function, physical activity, musculoskeletal symptoms, and sleep. Examining the effect of extremely short-term (one classroom lesson of 45 min) use of standing desks on executive function and stress levels, Tanaka and Noi (2022) indicate a higher number of correct answers in the Stroop test in the standing and switching conditions (mixing standing and sitting) than in the conventional condition without an excessive increase in stress levels. The use of standing desks for a period of 3 weeks significantly decreased musculoskeletal discomfort in the neck, shoulder, elbows, and lower back but had no overall effect on children’s daily physical activity levels (Ee et al., 2018). Regarding the mid-term effects, the introduction of height-adjustable standing desks for a period of 3–8 months showed a decrease in sitting behavior during school time (Ayala et al., 2016; Kidokoro et al., 2019), while improving executive function related to academic performance (Wick et al., 2018), but with no observed effect on sleep patterns (Rhee and Benden, 2020), musculoskeletal pain/discomfort, anthropometric measures, or blood pressure (Ayala et al., 2016). During a longer intervention period for the entire school year, this measure was shown to reduce sitting time along with the likelihood of discomfort in the neck and shoulders of the children over the full school year (Parry et al., 2019).

Overall, the current body of literature provides promising findings on the potential benefits of using dynamic office furniture to promote physical and psychological wellbeing in different populations, including office workers and students. However, more research, combining cognitive tests with neurophysiological and musculoskeletal pain/discomfort measurements, is needed to fully understand the potential benefits and limitations of dynamic office furniture and how it can be effectively implemented in different workplace and educational settings by exploring other modalities.

In this context, the previous work of our department has been pioneering in assessing the possible cognitive benefits of using dynamic office settings and the accompanying neurophysiological response. Particularly, because changes in the electroencephalogram (EEG) caused by movement-promoting and sensory-stimulating office setups serve as objective indicators of improved cognitive performance, psychophysiological states (such as arousal/alertness and stress reduction), and motivation (Henz and Schöllhorn, 2019), the primary focus of our previous and current research is on electrical brain activity measured by the EEG. Indeed, the EEG analysis of different frequency bands, including the theta (4–7.5 Hz), alpha (8–13 Hz), beta (13–30 Hz), and gamma (30–40 Hz) ranges, provides crucial insights into changes in cognitive performance and diverse psychophysiological states before, during, and after interventions in exercise-enhancing office environments. Theta activity is primarily associated with the consolidation of motor, somatosensory, and cognitive learning, while alpha activity occurs during both motor and cognitive learning processes, as well as during relaxation states. Beta activity, mainly observed pericentrally and frontally, reflects heightened alertness, and gamma activity is predominantly present during neuronal reorganization and high-information flow (Henz and Schöllhorn, 2019).

Furthermore, in light of the recently emphasized role of the autonomic nervous system in mental stress level and cognitive functioning (Kim et al., 2018; Forte et al., 2019), which is primarily corroborated by the correlation between reduced stress level, enhanced cognitive performance, and greater heart rate variability (HRV) in both the time and frequency domains during wakefulness (Forte et al., 2019), our department’s previous and ongoing investigations have also considered measuring HRV parameters and assessing mental exhaustion and wakefulness. In our recent study, we investigated the effects of working in a dynamic or static office environment for a period of 2 weeks on attentional and vigilance performance and on the corresponding perceived mental exhaustion and wakefulness, EEG brain oscillatory patterns, and HRV (Henz and Schöllhorn, 2019). The dynamic environment consisted of two desks that are height-adjustable, with one designed as a standing and the other as a sitting workstation, where participants were requested to change the workstation at randomly set time intervals (5–20 min). After 2 weeks of intervention, we observed increased attentional and vigilance performance in the dynamic group. This enhancement in cognitive performance was accompanied by greater subjectively perceived calm and wakefulness and higher activity of the parasympathetic nervous system, characterized by a decrease in the high-frequency band and an increase in the low-frequency band, as well as increased beta and gamma power in frontal and parietal areas during the attentional task and increased theta, alpha, and beta power in frontal, central, and parietal areas during the vigilance task. Additionally, EEG coherence increased using the dynamic office on various electrode pairs, including F3/F4, C3/C4, and P3/P4. We concluded that compared to the static environment, the dynamic one stimulates an optimum psychophysiological level of brain activation, wakefulness and mental stress level that mediates better attentional and vigilance performance (Henz and Schöllhorn, 2019).

However, given that the intervention sessions in this study were based on performing standardized everyday office tasks (e.g., document reading, e-mail correspondence, calculations) in individual settings, such a promising conclusion can only be valid for individual settings and should be interpreted with caution in a team-work setting, such as a meeting environment. Accordingly, these findings encourage further developments from individual dynamic office environments toward dynamic meeting environments consisting of height-adjustable meeting desks, mobile chairs, and floors that foster the whole team’s motor activities, which in turn stimulate the team’s brains and nervous system toward a state that is beneficial for cognitive performance during meeting settings. Since there is limited evidence about how a dynamic meeting environment affects teamwork and the neurophysiological responses to collaborative meeting tasks, it’s important to compare, in the same study, how short- and mid-term use of a dynamic meeting environment vs. a conventional meeting environment can affect cognitive performance, mental exhaustion and neurophysiological indicators during a real-world meeting. Interestingly, when evaluating mental exhaustion, such a study is encouraged to employ more objective measurement, such as the validated EEG-based mental workload index (EEG-MWI) (Holm et al., 2009), rather than using a subjective perceiving scale (e.g., subjectively perceived calm). Given that sleepiness has been demonstrated to result in cognitive performance deficits (Jackson and Van Dongen, 2011), it would also be interesting to measure sleepiness level along with cognitive performance. Similarly, considering that prolonged sitting posture is well known to be highly risky for the skeleton, joints, and muscles (Malinska et al., 2021), it would also be of interest to examine whether a dynamic meeting environment would reduce musculoskeletal discomfort compared to the conventional environment. Thus, the purpose of our study is to investigate the acute and mid-term effects of working in a dynamic meeting environment (Aeris^®^-meeting environment) on brain activity, cognitive performance, HRV, sleepiness, EEG-MWI, as well as local experienced discomfort (LED) in a team-based setting. By simulating meetings of a small team (i.e., three healthy adults) using different collaborative tasks under controlled laboratory conditions for a 2-week intervention period, our experiment was designed to test whether working in a specifically designed meeting environment, integrating a specifically designed meeting desk (Aeris^®^-meeting-desk) with active floor mats (Aeris^®^ muvmat) and ergonomic Aeris^®^ muvman chairs, would activate the brain and the nervous system toward a state that fosters improvements in attentional and vigilance performance, while reducing musculoskeletal discomfort, and mental exhaustion compared to meeting in a conventional static meeting environment. By combining brain and cardiac activity with cognitive performance measurements, this study seeks to provide a comprehensive understanding of the impact of dynamic meeting furniture on the short- and mid-term neuro-psycho-physiological responses of healthy adults. In order to account for potential confounding variables, we subjectively assessed physical activity behaviors, sitting time, and sleep patterns both 2 weeks before and during the intervention period. We hypothesized that the Aeris^®^ meeting environment would heighten parasympathetic nervous system and brain activity, particularly within the Alpha and Beta ranges, while improving attention and vigilance performance, decreasing mental workload and sleepiness, and alleviating local discomfort, with a deeper mid-term effect.

## 2 Materials and methods

### 2.1 Participants

The sample size was calculated *a priori* based on procedures suggested by Beck (2013) and using the software G*power (Faul et al., 2007). Values were set at 0.05 for α and 0.80 for power. Based on the studies of Henz and Schöllhorn (2019) and discussions between the authors, the effect size was estimated to be 0.5 (medium effect). The required sample size for this study was 22. Twenty-four healthy adults were recruited to voluntarily participate in this study. Participants were randomly assigned to either a control (i.e., conventional meeting environment, CG) or experimental (Aeris^®^ dynamic meeting-environment, DG) group with a 1:1 allocation as per a computer-generated randomization schedule stratified by sex and educational levels. After receiving a description of the protocol, potential risks and benefits of the study, participants gave their written consent to participate in this investigation. The demographic criteria for participant inclusion in the present study were as follows: all participants were aged between 18 and 29 years old, 18 of them were right-handed as assessed by the Edinburgh Handedness Inventory and 5 were ambiguous. Exclusion criteria included: current or a history of neurological and/or cardiovascular impairment, eye disorders, psychiatric illnesses, orthopedic diseases and/or muscular disorders, intake of medication that may have influenced EEG brain activity and/or HRV.

The study was conducted according to the Declaration of Helsinki, all subjects were naive as to the purpose of the study and were coded with numbers for anonymity of personal data. The study was approved by the local ethics committee of the University of Mainz.

### 2.2 Experimental design

**FIGURE 1:**
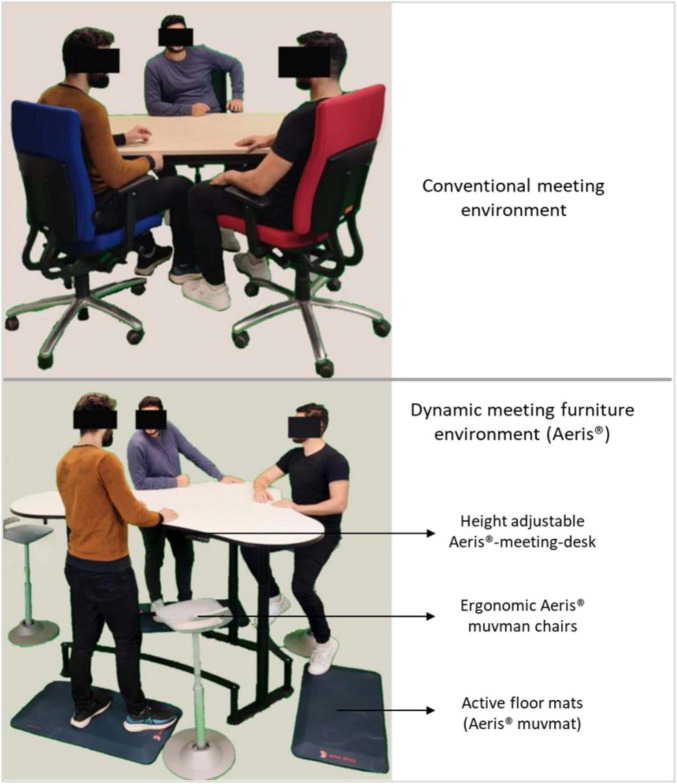
Conventional and dynamic meeting environments.

Using a between-subject design, the acute and mid-term effects of two different meeting environments were investigated. The experimental Aeris^®^-meeting environment represented the dynamic meeting environment and consisted of a configuration of a height-adjustable, specifically designed meeting desk (Aeris^®^-meeting-desk), three active floor mats (Aeris^®^ muvmat), and three ergonomic Aeris^®^ muvman chairs (Figure 1). The subjects were encouraged to change their position from sitting on the chair to standing on the mat every 30 min. The conventional meeting environment represented the conventional sitting-based environment and consisted of a normal, stable desk with static sitting furniture without active floor mats. The subjects were seated on a chair adjustable in height, ensuring only vertical movement, and had to keep their sitting position throughout the work (Figure 1).

**FIGURE 2:**
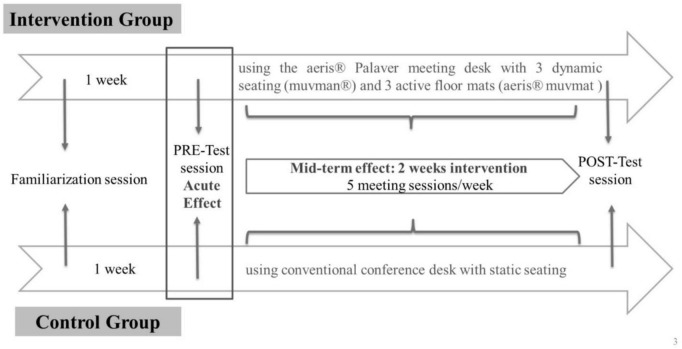
Study design.

After a familiarization session, participants completed two test sessions in the laboratory, namely the pre-intervention (PRE-test) and the post-intervention (POST-test) test sessions, separated by a 2-week intervention period (Figure 2). During the 2-week intervention period, participants were asked to attend, in a group of three interlocutors, 10 simulated meetings (5 meetings per week with a duration of 90 min for each meeting) consisting mainly of differential collaborative team asks/games (i.e., at least three tasks from different task categories) using either the Aeris^®^ Meeting (DG) or the conventional (CG) environment. These intervention meetings were simulated under controlled laboratory conditions, but with no programmed measurements. A more detailed content of the 90-min simulated meetings can be found in Annex 1.

The PRE- and POST- test sessions also consisted of simulated 90-min meetings and included specifically (i) an open discussion (20 min), (ii) a semantic word game (10 min), (iii) a cooperative game (20 min Hanabi game), (iv) a pop-quiz (10 min), and the guess the ball position game (10 min). To control the physical activity and sleep pattern before and during the intervention, participants completed the short form of the International Physical Activity Questionnaire (IPAQ-SF) and responded to questions 4 (self-estimation of sleep duration) and 9 (self-estimation of sleep quality) from the Pittsburgh Sleep Quality Index (PSQI) questionnaire at the start of each test session. Various measurements were then conducted during both test sessions. Indeed, spontaneous EEG and HRV activities (duration: 2 min) were recorded simultaneously, in sitting position with eyes open, at the beginning of each test session and immediately following the 90-min differential collaborative tasks. Additionally, before and after the simulated 90-min meeting, attentional and vigilance cognitive performances were recorded during the short-term attention D2-R test and the psychomotor vigilance task (PVT), respectively. At the end of each cognitive test boot, participants’ subjective levels of sleepiness were assessed using the Karolinska Sleepiness Scale (KSS); and their mental workload was assessed using the EEG mental workload index (EEG-MWI). Finally, subjective discomfort was determined using the Local Experienced Discomfort (LED). All these measures were performed simultaneously for the three participating interlocutors at each test session. All test sessions were performed at the same time of day (in the afternoon), as previously suggested by Ammar et al. (2017) to minimize the effect of diurnal biological variations (Ammar et al., 2017). The measurements were carried out under laboratory conditions. Changes in brightness, volume, and temperature were standardized or kept to a minimum.

By using a PRE- and POST-test design, it was possible to make statements about the short- and mid-term effects of using the Aeris^®^-meeting environment. Particularly, the data from the pre- and post-simulated 90-min meetings recorded during the first test session (PRE-test session) was used to determine the acute effects. While data from both PRE- and POST- test sessions (intercepted by the 2-week intervention period) was compared to determine the mid-term effects.

### 2.3 Measurements

#### 2.3.1 Physical activity and sleep pattern

The IPAQ-SF had been extensively validated in different cultures and populations (Craig et al., 2003). The total weekly PA (MET-min ⋅ week-1) was estimated by multiplying the reported weekly time for each IPAQ-SF item (vigorous intensity, moderate intensity, and walking) by their respective Metabolic Equivalent of Task (MET) values (Lee et al., 2011). We utilized the original MET values recommended by the official IPAQ guidelines for young and middle-aged adults (18–65 years old): vigorous PA = 8.0 METs, moderate PA = 4.0 METs, and walking = 3.3 METs. Following the IPAQ scoring protocol, participants in the study were categorized into three groups based on their MET–min/wk, which represents the cumulative sum of walking, moderate-intensity physical activities, and vigorous-intensity physical activities: lowly active (<600 MET–min/wk), moderately active (600 MET–min/wk ≤ PA < 3,000 MET–min/wk), and highly active (≥3,000 MET–min/wk).^1^

Additionally, to evaluate subjective sleep quality and quantity during the week preceding each test session, participants responded to question 4 (hours of actual sleep per night) and question 6 (rating of overall sleep quality: very good, fairly good, fairly bad, or very bad) from the PSQI questionnaire. Similarly, PSQI has undergone extensive validation across diverse cultures and populations (Buysse et al., 1989; Farrahi Moghaddam et al., 2012).

#### 2.3.2 Mobile EEG headset

**FIGURE 3:**
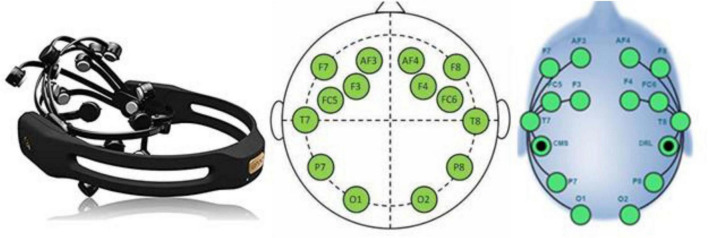
The Emotiv™ Epoc+.

Electroencephalogram can monitor subjects’ cognitive states by directly capturing electrical brain activity. The emergence of low-cost and wireless EEG makes it affordable and easier to use. In the present study, the EEG data was recorded simultaneously from the three tested interlocutors using the wireless EEG headset Emotiv™ Epoc + (version 2020). Emotiv EPOC is a lightweight, low-cost, high-resolution, wireless, multi-channel headset that uses an array of 14 sensors to detect electrical signals generated by the brain (Sánchez-Reolid et al., 2018). This headset is composed of 14 saline electrodes organized according to the 10–20 system (Jasper, 1958) (F3, F4, AF3, AF4, F7, F8, FC5, FC6, O1, O2, T7, T8, P7, P8) and 2 references on parietal sites (P3 and P4) (Figure 3). The 10–20 system is based on the relationship between the electrode location and the sub-regions of the cerebral cortex. Each point on the system indicates a possible position for the electrode, with a letter to identify the lobes and a number to identify the hemisphere. The 10–20 system ensures accurate electrode placement, enabling researchers to obtain reliable EEG signals. Spontaneous EEG signals were continuously registered using the Emotiv™ Epoc software with a down sampling rate of 128 Hz after filtering.

Emotiv Epoc + headsets were connected to the Emotiv Pro software, which first checks the contact quality of the electrodes. During data acquisition with this system, the contact quality is given as 0 = no contact, 1 = low contact quality, 2 = medium contact quality, 3 = good contact quality, and 4 = excellent contact quality. All electrodes were kept on quality level 4 during the experiment.

**FIGURE 4:**
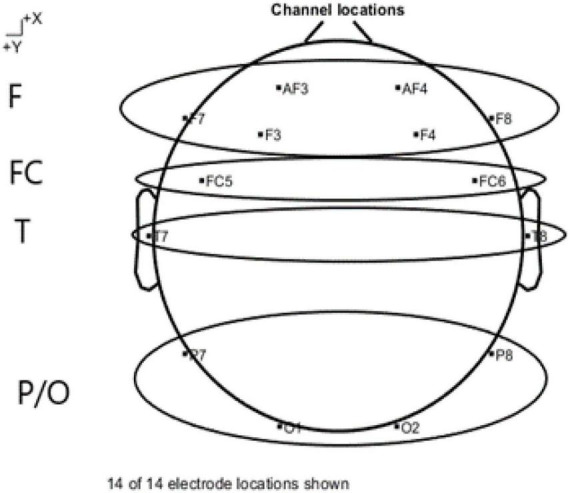
Scalp location.

Four scalp location (Figure 4), frontal (F3, F4, AF3, AF4, F7, F8), fronto-central (FC5, FC6), temporal (T7, T8) and parietal-occipital (O1, O2, P7, P8) were chosen to gather the signals from the 14 electrodes (Lord and Opacka-Juffry, 2016; Wind et al., 2020).

Once the raw signals were recorded in Emotiv Pro, we exported them to MATLAB (EEGLAB) for processing the signals.

#### 2.3.3 EEG data pre-processing and analysis

The data were visually inspected to remove noisy segments related to electrode placement or technical disturbances. A bandpass filter was set by default on the Emotiv™ system to 0.2–45 Hz. Digital notch filters at 50 and 60 Hz were applied to remove power-line interferences, which often interfere with electrophysiological signals. Independent Component Analysis (ICA) was performed using Matlab-based EEGLAB-toolbox 2019 from the University of California San Diego (Delorme and Makeig, 2004), in order to deduce recurrent movement from the signal like eye blinks, muscle activity, or channel noise. Impaired electrodes were interpolated using a spherical spline interpolation method (Perrin et al., 1989). A final visual inspection of the time series and the power spectrum was conducted to remove remaining artifacts. Spectral power was calculated for each testing session using Fast Fourier Transformation with a Hanning window, a window size of 4,096 samples (4 s), and a window overlap of 50% for the theta (3.5–7.5 Hz), alpha (7.5–12.5 Hz), beta (12.5–30 Hz) and gamma (30–40 Hz) bands. Oscillations above 40 Hz were not usable because of the internal band-pass filter. Band ranges were chosen according to previous studies that involved movement and EEG (Henz et al., 2018; Wind et al., 2020; John et al., 2022). The power spectrum was computed for each test session, frequency band, and brain lobe.

#### 2.3.4 EEG-based mental workload index

Electroencephalogram signals are widely adopted by researchers to measure mental workload, with previous findings showing increased theta band activity of the frontal lobe and decreased alpha band activity of the parietal lobe associated with increased mental workload (Lean and Shan, 2012). By comparing several EEG signal indexes, Holm et al. (2009) found the ratio of frontal theta power and parietal alpha power is more sensitive in reflecting mental workload. Considering the configuration of the 14-channel EEG headset, in this study, we used the mental workload index calculated by Wang et al. (2019) using the following equation.


$$Mentalworkloadindex=\frac{Averagefrontalthetapower}{Averageparietalalphapower}=\frac{F\,3\,T\,h\,e\,t\,a\,R\,P+F\,4\,T\,h\,e\,t\,a\,R\,P}{P\,7\,A\,l\,p\,h\,a\,R\,P+P\,8\,A\,l\,p\,h\,a\,R\,P}$$

A large mental workload index represents a high mental workload.

#### 2.3.5 HRV measurements

Heart rate variability (HRV) is a non-invasive measure of the variability in time intervals between consecutive heartbeats, which reflects in part the functioning of the autonomic nervous system. Additionally, HRV is becoming an increasingly important parameter to objectively quantify the stress level of subjects (Kim et al., 2018). In this study, HRV parameters were measured using a Polar H10 HR monitor with a Pro Strap (Polar Electro Oy, Kempele, Finland). The Polar H10 system measures the electrical signal of the heart in the form of a 1-channel electrocardiogram (ECG) signal using two dry electrodes. From the ECG signal, the R-peaks are detected to derive the RR interval time series. Both the ECG quality (Skála et al., 2022) and the quality of the derived RR-intervals (Gilgen-Ammann et al., 2019; Schaffarczyk et al., 2022) using the Polar H10 were previously compared to the gold standard ECG measurement using a 12-channel medical-grade ECG device and found excellent.

#### 2.3.6 Measurement and analysis

Prior to the test sessions, the Polar H10 electrodes were moistened with room-temperature water prior to being placed on the xiphoid process of the sternum with the chest strap fitted around the participant’s chest (just below the chest muscles) (Ammar et al., 2021). The Android app “Polar Sensor Logger” (Happonen, 2019) was used to record the RR-intervals for all participants simultaneously. Two minutes of resting in an upright sitting position were recorded. The recorded data was later imported and analyzed in Kubios HRV Standard 3.5.0 analysis software (Tarvainen et al., 2014). Using Kubios, artifact removal and detrending were performed as necessary preprocessing steps. Therefore, RR intervals that were larger or smaller than a set threshold compared to the local average were corrected by replacing the identified artifacts with interpolated values using cubic spline interpolation. The threshold was adjusted individually but was generally in the range of 0.25–0.35 s (low or medium threshold). Afterward, the smoothness priors detrending method (Tarvainen et al., 2002) was used to avoid the effect of slow non-stationary trends in the analysis.

Using the preprocessed RR intervals, several time- and frequency-domain parameters were calculated. From the time domain, we chose the most often used parameters for short-term analysis, which are the mean (MeanRR), the standard deviation (SDNN) and the root mean square of successive differences (RMSSD) of the RR-intervals. For calculating the frequency parameters, the time series was resampled to 4 Hz, and the FFT spectrum was derived using Welch’s periodogram. The low frequency (LF, 0.04–0.15 Hz) and high frequency (HF, 0.15–0.4 Hz) power (n.u.) and the LF/HF ratio were computed from the FFT spectrum. Furthermore, we calculated the coefficient of variation (CoV) by the formula CoV = 100 * SDNN/MeanRR. The CoV tries to minimize the mathematical dependence of the standard deviation from the mean by normalization.

#### 2.3.7 Assessment of attentional and vigilance performance

The level of concentrated visual attention of participants was assessed using the d2-R test. The d2 test is an internally consistent and valid measure of visual scanning accuracy and speed with high test-retest reliability coefficients for all parameters, ranging from 0.95 to 0.98 (Bates and Lemay, 2004). This test consists of 14 rows with 47 characters per line. The characters used are the letters “d” or “p,” with a total of one to four dashes above and below each letter. Participants were asked to scan each line following a standard order and cross out only the characters containing the letter “d” with two dashes during the given 20 s per line. To maintain measurement accuracy, the first and last trials were excluded from the analysis, as proposed by Brickenkamp (1962, 1966). After completion of the d2-R test, two variables were calculated: concentration performance (CP) and total number of errors (E). CP was calculated as the number of correctly marked d2-symbols minus the total number of E. The total number of E was assessed by adding the end total values of omission error and mistake of confusion.

Vigilance performance was assessed by the 5-min Psychomotor Vigilance Task (PVT), a short form of the original 10-min PVT by Dinges and Powell (1985). The PVT is an often used and validated assessment of one’s neurocognitive capacities pertaining to sustained alertness, especially during fatigue (Loh et al., 2004; Arsintescu et al., 2019; Thompson et al., 2022). In this test, subjects have the task of reacting to stimuli (a red dot) appearing on an otherwise blank screen. The interstimulus interval varied from 2 to 10 s, with each PVT consisting of multiple trials (80–100 stimulus-response events). A participant’s response was recorded when the participant pressed the spacebar on their computer. The reaction time (RT) is the time between the appearance of the stimulus and the response of the participant. If a click is made before the stimulus comes up, the click is noted as a false start. RTs bigger than 500 ms were counted as missed lapses. The used outcome metrics include mean RT, mean 1/RT (Reciprocal response time; RRT), fastest 10% RT (Optimum response time; OPT-RT), and number of lapses (RT > 500 ms). A PVT response was considered valid if the RT was > 100 ms (responses with an RT < 100 ms were counted as false starts). High test-retest intraclass correlation coefficients were recently shown for the mean RT (ICCs = 0.79) and fastest RT% (ICCs = 0.83) variables of the 5 min-PVT in working-aged females (Thompson et al., 2022). Similarly, in classroom setting, high reliability was found for mean RT (ICCs = 0.84), while number of lapses showed moderate ICCs (0.59) among male students (Wilson et al., 2010).

#### 2.3.8 KSS

The self-reported Karolinska Sleepiness Scale (KSS) has proven to be a robust and reliable tool for assessing the subjective level of sleepiness at a particular time during the day, as evidenced by high intraclass correlation coefficients (ICCs). Notably, in a study by Van Dongen et al. (2004), the ICC for the KSS was 0.90 between two bouts of 36 h of total sleep deprivation, indicating remarkable individual stability across different conditions as well as KSS’s superior reliability and consistency compared to other scales, such as the Karolinska Drowsiness Score (KDS) (Akerstedt et al., 2014). Moreover, KSS was validated against alpha and theta electroencephalographic (EEG) activity as well as slow eye movement electrooculographic (EOG) activity (Akerstedt and Gillberg, 1990) and has been widely used and provided reasonable results in studies of attention and performance (Gillberg et al., 1994, 1996; Reyner and Horne, 1998; Kräuchi et al., 2006), making it a valuable choice for our study. On this scale, subjects indicate which level best reflects the psycho-physical state they experienced in the last 10 min. KSS is a 9-point scale (1 = extremely alert, 3 = alert, 5 = neither alert nor sleepy, 7 = sleepy – but no difficulty remaining awake, and 9 = extremely sleepy – fighting sleep) (Akerstedt and Gillberg, 1990; Fatek, 2021).

#### 2.3.9 LED

The LED scale allows participants to express their physical discomfort during 90-min tasks on a scale of 0 (no complaints at all) to 10 (extreme amount of complaints) points (Corlett and Bishop, 1976). The use of this scale is widely accepted in ergonomics research (van der Schatte Olivier et al., 2009). In the present study, we focused on four local body parts (i.e., neck, shoulder, upper back, and lower back).

### 2.4 Data analysis

The neurophysiological data are subjected to a preliminary evaluation [artifact correction, independent component analysis (ICA)] with the Matlab-based EEG software EEG Lab and a subsequent in-depth evaluation using spectral analysis.

Mean and standard deviation (SD) values were calculated for each variable. The Shapiro-Wilk W-test was used to check if variables did not statistically deviate from their normal distribution. Data are then processed using inferential statistical methods (e.g., multivariate analysis of variance). To estimate the meaningfulness of significant differences, effect sizes were calculated as partial eta-squared (ηp2) for the main effects and the interaction between them and as Cohen’s d (d) for the paired comparison. Values of 0.01, 0.06, and 0.13 for partial eta-squared and 0.2, 0.5, and > 0.8 for Cohen’s d represent small, moderate, and large effect sizes, respectively. To estimate the magnitude of significant differences, difference (Δ) or percent difference [Δ (%)] scores were calculated as follows: Δ = (post value - pre value); Δ (%) = [(post value–pre value)/(pre value)] × 100.

The acute effects of using the dynamic office furniture (Aeris^®^-meeting-environment) were tested, based on the data of the pre-intervention test session, by comparing the values at post-90 min meeting vs. pre-90 min meeting in both groups using a two-way ANOVA [2 groups × 2 conditions (pre-post 90 min)]. Δ or Δ (%) from pre- to post-90 min meetings were also compared between groups during the pre-intervention session using an independent *t*-test or Mann-Whitney test.

The mid-term effects were first determined by comparing the pre-90 min meeting values recorded at the pre-intervention test session (PRE-test) vs. post-intervention test session (POST-test) in both groups using two-way ANOVA [2 groups × 2 conditions (pre-post intervention)].

Additionally, the Δ or Δ (%) from pre- to intervention were also compared between the PRE-test and POST-test in both groups to evaluate the mid-term effects of the intervention using a two-way ANOVA [2 groups × 2 conditions (pre-post intervention)].

To control for possible significant differences between the control and the dynamic groups in terms of PA behaviors and sleep patterns both before and during the intervention, a two-way ANOVA [2 groups × 2 periods (pre- and during-intervention)] was conducted. Significance was accepted for all analyses at the level of *p* < 0.05. Exact *p*-values have been given.

## 3 Results

### 3.1 Participants characteristics

**FIGURE 5:**
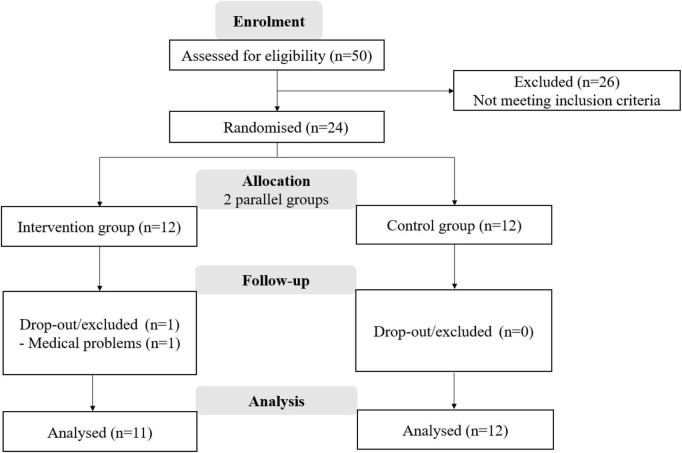
CONSORT flow diagram.

Fifty participants were screened, and 24 were deemed eligible to participate in the Aeries intervention (16 females and 8 males, age: 25.2 ± 3.1 years old, weight: 68.6 ± 5.8 kg, height: 1.7 ± 0.11 m). Twelve were allocated to the control group and twelve to the dynamic group. One participant in the dynamic group dropped out during the training intervention due to a medical problem (SARS-COV2). Twenty-three healthy adults completed the study. The dynamic group used the Aeris Palaver standing table with active floor mats and Muvman chairs, and the control group used the conventional conference table with static seating. In the present results section, data from these 23 participants was included in the final analysis. Figure 5 shows the flowchart of the subject’s screening and participation.

### 3.2 Participants’ physical activity behaviors and sleep patterns

Table 1 shows the estimated total weekly PA (i.e., MET values) and daily sitting time and sleep duration of the control and dynamic groups both before and during the intervention. ANOVA analysis showed no significant main effect of “groups” and “periods” as well as “group” × “periods” interaction for all tested parameters. Similarly, *post hoc* analysis revealed no significant differences between groups at both before and during intervention (*p* > 0.05) in all tested parameters. Furthermore, it’s worth noting that 75–81% of the participants in both groups reported their sleep quality to be fairly to very good, both before and during the intervention. These results suggest that the potential confounding effects of PA and sleep pattern variables during the dynamic meeting intervention are expected to be minimal in this study.

**TABLE 1:** Participants’ physical activity behaviors, sitting times and sleep duration both before and during the intervention.

	Before intervention		During intervention		2 ways ANOVA		
	Control group	Dynamic group	Control group	Dynamic group	Interaction Groups × Periods (F, *p*-value, η p^2^)	Groups effect (F, *p*-value, η p^2^)	Periods effect (F, *p*-value, η p^2^)
Weekly PA (MET-min ⋅ week-1)	2403.47 ± 1516.26	2348.37 ± 1555.47	2733.91 ± 1504.75	2094.17 ± 1223.63	(*F* = 0.72, *p* = 0.41, ηp^2^ = 0.03)	(*F* = 0.01, *p* = 0.94, ηp^2^ = 0.0002)	(*F* = 1.01, *p* = 0.33, ηp^2^ = 0.05)
Daily sitting time (hours)	5.5 ± 2.31	4.91 ± 1.81	4.54 ± 1.70	4.75 ± 1.71	(*F* = 0.56, *p* = 0.46, ηp^2^ = 0.03)	(*F* = 0.93, *p* = 0.35, ηp^2^ = 0.04)	(*F* = 0.13, *p* = 0.72, ηp^2^ = 0.01)
Sleep duration (hours)	6.9 ± 1.60	7.36 ± 1.36	6.17 ± 1.71	6.08 ± 1.69	(*F* = 0.39, *p* = 0.54, ηp^2^ = 0.02)	(*F* = 3.79, *p* = 0.07, ηp^2^ = 0.15)	(*F* = 0.17, *p* = 0.68, ηp^2^ = 0.01)

### 3.3 Acute effects of using Aeris^®^-meeting-environment

#### 3.3.1 Resting EEG

The acute effects of using Aeris^®^-meeting-environment on resting brain activity are presented in Table 2 for theta, alpha, beta and gamma frequencies, respectively.

**TABLE 2:** Acute effect of using Aeris^®^-meeting-environment on EEG signal (μV) for theta, alpha, beta, and gamma frequencies recorded at the frontal, fronto-central, temporal, and occipito-parietal regions.

	Pre-90 min		Post-90 min		2 ways ANOVA		
	Control group	Dynamic group	Control group	Dynamic group	Interaction Group × Meeting-session (F, *p*-value, η p^2^)	Group effect (F, *p*-value, η p^2^)	Meeting-session effect (F, *p*-value, η p^2^)
**Theta frequency (mean ± SD)**							
Frontal region	4.60 ± 1.21	4.75 ± 2.33	5.97 ± 1.61	6.00 ± 1.32	(*F* = 0.01, *p* = 0.91, ηp^2^ = 0.0003)	(*F* = 0.03, *p* = 0.86, ηp^2^ = 0.0008)	(*F* = 6.89, *p* = 0.01, ηp^2^ = 0.15)
Fronto-central region	4.06 ± 1.25	4.55 ± 1.41	5.20 ± 1.98	5.48 ± 1.52	(*F* = 0.05, *p* = 0.82, ηp^2^ = 0.001)	(*F* = 0.64, *p* = 0.43, ηp^2^ = 0.02)	(*F* = 4.71, *p* = 0.04, ηp^2^ = 0.11)
Temporal region	1.78 ± 2.43	2.82 ± 1.39	2.95 ± 1.41	3.53 ± 1.62	(*F* = 0.18, *p* = 0.68, ηp^2^ = 0.004)	(*F* = 2.22, *p* = 0.14, ηp^2^ = 0.05)	(*F* = 3.02, *p* = 0.09, ηp^2^ = 0.07)
Parieto-occipital region	3.10 ± 2.46	3.35 ± 1.39	4.14 ± 1.44	4.19 ± 1.73	(*F* = 0.03, *p* = 0.86, ηp^2^ = 0.0008)	(*F* = 0.07, *p* = 0.79, ηp^2^ = 0.002)	(*F* = 2.89, *p* = 0.10, ηp^2^ = 0.07)
**Alpha frequency (mean ± SD)**							
Frontal region	5.32 ± 2.62	3.90 ± 2.47	6.14 ± 2.11	5.34 ± 2.38	(*F* = 0.19, *p* = 0.67, ηp^2^ = 0.01)	(*F* = 2.35, *p* = 0.13, ηp^2^ = 0.06)	(*F* = 2.41, *p* = 0.13, ηp^2^ = 0.06)
Fronto-central region	4.86 ± 2.53	4.36 ± 1.86	5.68 ± 2.25	5.34 ± 2.01	(*F* = 0.02, *p* = 0.90, ηp^2^ = 0.0004)	(*F* = 0.39, *p* = 0.53, ηp^2^ = 0.01)	(*F* = 1.83, *p* = 0.18, ηp^2^ = 0.04)
Temporal region	2.52 ± 3.00	2.56 ± 1.93	3.35 ± 1.71	3.33 ± 2.01	(*F* = 0.003, *p* = 0.96, ηp^2^ = 0.0001)	(*F* = 0.0001, *p* = 0.99, ηp^2^ = 0.00001)	(*F* = 2.27, *p* = 0.15, ηp^2^ = 0.10)
Parieto-occipital region	5.66 ± 3.56	4.85 ± 2.92	6.11 ± 1.95	5.93 ± 3.23	(*F* = 0.30, *p* = 0.59, ηp^2^ = 0.02)	(*F* = 0.19, *p* = 0.67, ηp^2^ = 0.01)	(*F* = 1.76, *p* = 0.20, ηp^2^ = 0.08)
**Beta frequency (mean ± SD)**							
Frontal region	0.01 ± 1.61	−0.48 ± 2.40	0.41 ± 1.94	0.17 ± 1.26	(*F* = 0.05, *p* = 0.82, ηp^2^ = 0.001)	(*F* = 0.43, *p* = 0.52, ηp^2^ = 0.01)	(*F* = 0.89, *p* = 0.35, ηp^2^ = 0.02)
Fronto-central region	0.22 ± 1.29	−0.27 ± 1.45	0.22 ± 2.54	0.01 ± 1.14	(*F* = 0.07, *p* = 0.79, ηp^2^ = 0.002)	(*F* = 0.46, *p* = 0.50, ηp^2^ = 0.01)	(*F* = 0.07, *p* = 0.79, ηp^2^ = 0.002)
Temporal region	−1.91 ± 3.49	−1.66 ± 1.66	−1.59 ± 1.95	−1.74 ± 0.73	(*F* = 0.08, *p* = 0.77, ηp^2^ = 0.002)	(*F* = 0.01, *p* = 0.94, ηp^2^ = 0.0002)	(*F* = 0.03, *p* = 0.86, ηp^2^ = 0.001)
Parieto-occipital region	−0.92 ± 2.86	−1.13 ± 2.09	−1.16 ± 2.01	−1.02 ± 1.26	(*F* = 0.08, *p* = 0.78, ηp^2^ = 0.002)	(*F* = 0.003, *p* = 0.96, ηp^2^ = 0.0001)	(*F* = 0.01, *p* = 0.92, ηp^2^ = 0.0002)
**Gamma frequency (mean ± SD)**							
Frontal region	−1.57 ± 1.73	−2.82 ± 2.30	−1.69 ± 2.01	−2.55 ± 1.48	(*F* = 0.12, *p* = 0.73, ηp^2^ = 0.003)	(*F* = 3.34, *p* = 0.08, ηp^2^ = 0.08)	(*F* = 0.02, *p* = 0.89, ηp^2^ = 0.001)
Fronto-central region	−0.99 ± 1.53	−2.18 ± 1.48	−1.61 ± 2.67	−2.54 ± 1.83	(*F* = 0.05, *p* = 0.83, ηp^2^ = 0.001)	(*F* = 3.16, *p* = 0.08, ηp^2^ = 0.07)	(*F* = 0.68, *p* = 0.41, ηp^2^ = 0.02)
Temporal region	−2.85 ± 3.84	−3.42 ± 1.36	−3.01 ± 1.89	−3.87 ± 0.99	(*F* = 0.04, *p* = 0.84, ηp^2^ = 0.001)	(*F* = 0.98, *p* = 0.33, ηp^2^ = 0.02)	(*F* = 0.17, *p* = 0.68, ηp^2^ = 0.004)
Parieto-occipital region	−2.56 ± 3.01	−3.50 ± 1.77	−3.27 ± 2.04	−3.77 ± 1.33	(*F* = 0.11, *p* = 0.74, ηp^2^ = 0.003)	(*F* = 1.20, *p* = 0.28, ηp^2^ = 0.03)	(*F* = 0.55, *p* = 0.46, ηp^2^ = 0.01)

A statistically significant main effect of meeting-session was only found for theta frequency at the frontal FP-F (*p* = 0.01, np2 = 0.15) and Fronto-central FC (*p* = 0.04, np2 = 0.11) region with no significant group effect or “group” × “meeting-session” interaction. This main effect indicates slight activation increases in the theta frequency band in frontal and central regions following the 90-min meeting in both CG and DG groups.

However, as indicated by the *post hoc* analysis, there were no significant differences between the groups. Similarly, no significant difference was found between groups in terms of Δ activation from pre- to post-90 min meeting. There was no significant main effect of “group,” “meeting-session” or interaction “group” × “meeting-session” in alpha, beta, and gamma frequency bands (*p* > 0.05).

#### 3.3.2 Cognitive performances

##### 3.3.2.1 Attentional performance (d2)

The acute effect of using Aeris^®^-meeting-environment on d2 concentration performance (CP) and total number of errors (E) are presented in Table 2.

A significant main effect of meeting-session was only found for CP (*p* = 0.03, np2 = 0.12), with no significant group effects or “group” × “meeting-session” interaction. This significant main effect reflects slight, non-significant increases in concentration performance following the 90-min meeting in both CG and DG groups. However, as indicated by the *post hoc* analysis, there were no significant differences between the groups. Similarly, no significant difference was found between groups in terms of Δ concentration performance from pre- to post-90 min meeting. There were no significant main effects of “group,” “meeting-session” or interaction “group” × “meeting-session” in the total number of errors (*p* > 0.05).

##### 3.3.2.2 PVT

The acute effect of using Aeris^®^-meeting-environment on PVT vigilance performance are presented in Table 2.

ANOVA analysis showed no significant main effect of “meeting-session” and “group” as well as no “group” × “meeting-session” interaction for all tested parameters (RT, RRT, OPT-RT, and N-Lapses). *Post hoc* analysis showed a significant increase (*p* = 0.04, *d* = 0.10) in N-lapses from pre- to post- 90 min meetings, only in the CG (+ 2.5 ± 3 lapses), while the DG showed to maintain this performance (*p* > 0.05). For the remaining parameters, there were no significant changes from pre- to post- meeting as well as no significant differences between groups in any of the measurement points (*p* > 0.05). Similarly, no significant difference was found between groups in terms of Δ vigilance performances from pre- to post-90 min meeting.

#### 3.3.3 Heart rate variability parameters

The acute effect of using Aeris^®^-meeting-environment on HRV’s time and frequency domains parameters are presented in Table 2. The ANOVA showed no significant main effect of “meeting-session” and “group” as well as no “group” × “meeting-session” interaction for all tested parameters. *Post hoc* analysis showed no significant changes from pre- to post-test meeting as well as no significant differences between groups at any of the measurement points (*p* > 0.05). Similarly, no significant difference was found between groups in terms of Δ HRV from pre- to post-90 min meeting.

#### 3.3.4 Sleepiness and mental workload

The acute effect of using Aeris^®^-meeting-environment on sleepiness (KSS) and mental workload (EEG-MWI) are presented in Table 3. The ANOVA showed no significant main effect of “group,” “meeting-session” or interaction “group” × “meeting-session” in KSS and EEG-MWI (*p* > 0.05). A significant difference between CG and DG group was only found in KSS values recorded at post-90 min meeting with higher KSS values in CG (*p* = 0.05, *d* = −0.84). No significant difference was found between groups in terms of Δ KSS or EEG-MWI from pre- to post-90 min meeting.

**TABLE 3:** Acute effect of using Aeris^®^-meeting-environment on d2 attentional performance, PVT vigilance performance, HRV’s time and frequency domain parameters, and sleepiness (KSS) and mental workload (EEG-MWI).

		Pre-90 min		Post-90 min		2 ways Anova		
		Control Group	Dynamic Group	Control Group	Dynamic Group	Interaction Group × Meeting-session (F, *p*-value, η p^2^)	Group effect (F, *p*-value, η p^2^)	Meeting-session effect (F, *p*-value, η p^2^)
**Cognitive performance**								
D2	CP	156.2 ± 21.38	169.64 ± 34.43	175.8 ± 24.59	193.36 ± 39.32	(*F* = 0.05, *p* = 0.83, ηp^2^ = 0.001)	(*F* = 2.59, *p* = 0.12, ηp^2^ = 0.06)	(*F* = 5.07, *p* = 0.03, ηp^2^ = 0.12)
	E	20 ± 9.71	23.5 ± 14.48	15.7 ± 6.57	20.8 ± 14.23	(*F* = 0.03, *p* = 0.87, ηp^2^ = 0.001)	(*F* = 0.97, *p* = 0.33, ηp^2^ = 0.03)	(*F* = 1.02, *p* = 0.32, ηp^2^ = 0.03)
PVT	RT (ms)	360.73 ± 29.54	343.96 ± 25.00	358.5 ± 25.68	355.73 ± 37.65	(*F* = 0.60, *p* = 0.44, ηp^2^ = 0.02)	(*F* = 1.18, *p* = 0.29, ηp^2^ = 0.03)	(*F* = 0.28, *p* = 0.60, ηp^2^ = 0.01)
	RRT (sec^–1^)	2.78 ± 0.21	2.77 ± 0.17	2.91 ± 0.23	2.83 ± 0.27	(*F* = 0.27, *p* = 0.61, ηp^2^ = 0.01)	(*F* = 2.00, *p* = 0.17, ηp^2^ = 0.05)	(*F* = 0.59, *p* = 0.45, ηp^2^ = 0.01)
	OPT-RT (ms)	303.18 ± 29.64	298.09 ± 23.72	300.18 ± 23.87	305.31 ± 28.00	(*F* = 0.41, *p* = 0.53, ηp^2^ = 0.01)	(*F* = 0.000, *p* = 0.99, ηp^2^ = 0.000)	(*F* = 0.07, *p* = 0.79, ηp^2^ = 0.002)
	N-Lapses	1.36 ± 1.29	2.82 ± 2.96	3.82 ± 3.25a	2.72 ± 3.10	(*F* = 2.33, *p* = 0.14, ηp^2^ = 0.06)	(*F* = 0.05, *p* = 0.83, ηp^2^ = 0.001)	(*F* = 2.01, *p* = 0.16, ηp^2^ = 0.05)
**HRV’s time domain**								
Mean HR (bpm)		76.23 ± 14.43	69.68 ± 6.82	71.33 ± 8.04	67.66 ± 6.78	(*F* = 0.25, *p* = 0.62, ηp^2^ = 0.01)	(*F* = 3.14, *p* = 0.08, ηp^2^ = 0.07)	(*F* = 1.44, *p* = 0.24, ηp^2^ = 0.04)
Mean RR (ms)		809.27 ± 132.90	868.83 ± 87.35	850.88 ± 94.71	895.144 ± 92.32	(*F* = 0.06, *p* = 0.81, ηp^2^ = 0.002)	(*F* = 2.77, *p* = 0.10, ηp^2^ = 0.07)	(*F* = 1.19, *p* = 0.28, ηp^2^ = 0.03)
SDNN (ms)		39.42 ± 11.33	45.56 ± 16.09	40.84 ± 9.81	47.88 ± 18.29	(*F* = 0.01, *p* = 0.92, ηp^2^ = 0.0002)	(*F* = 2.34, *p* = 0.13, ηp^2^ = 0.06)	(*F* = 0.19, *p* = 0.67, ηp^2^ = 0.01)
RMSSD (ms)		37.15 ± 11.33	45.78 ± 18.57	38.52 ± 11.70	49.40 ± 20.87	(*F* = 0.05, *p* = 0.82, ηp^2^ = 0.001)	(*F* = 4.01, *p* = 0.05, ηp^2^ = 0.09)	(*F* = 0.26, *p* = 0.61, ηp^2^ = 0.01)
**HRV’s Frequency domains and covariance**								
LF (ms^2^)		49.96 ± 17.62	46.77 ± 19.83	51.55 ± 19.03	49.49 ± 24.87	(*F* = 0.01, *p* = 0.93, ηp^2^ = 0.0002)	(*F* = 0.18, *p* = 0.67, ηp^2^ = 0.01)	(*F* = 0.12, *p* = 0.73, ηp^2^ = 0.003)
HF (ms^2^)		50.03 ± 17.61	53.19 ± 19.83	48.44 ± 19.03	50.47 ± 24.85	(*F* = 0.01, *p* = 0.93, ηp^2^ = 0.0002)	(*F* = 0.18, *p* = 0.68, ηp^2^ = 0.004)	(*F* = 0.12, *p* = 0.73, ηp^2^ = 0.003)
LF/HF ratio		1.27 ± 0.88	2.04 ± 4.08	1.33 ± 0.77	7.08 ± 19.72	(*F* = 0.67, *p* = 0.42, ηp^2^ = 0.016)	(*F* = 1.15, *p* = 0.29, ηp^2^ = 0.03)	(*F* = 0.7, *p* = 0.41, ηp^2^ = 0.02)
CoV (%)		0.05 ± 0.01	0.05 ± 0.02	0.05 ± 0.01	0.06 ± 0.02	(*F* = 0.01, *p* = 0.92, ηp^2^ = 0.0002)	(*F* = 1.13, *p* = 0.29, ηp^2^ = 0.03)	(*F* = 0.08, *p* = 0.77, ηp^2^ = 0.002)
**Sleepiness and mental workload**								
KSS		4.75 ± 2.45	4.36 ± 1.80	5.75 ± 2.30	4.18 ± 1.17b	(*F* = 0.99, *p* = 0.33, ηp^2^ = 0.02)	(*F* = 2.70, *p* = 0.11, ηp^2^ = 0.06)	(*F* = 0.47, *p* = 0.50, ηp^2^ = 0.01)
EEG-MWI		0.65 ± 1.72	2.90 ± 2.56	3.44 ± 7.22	3.98 ± 5.53	(*F* = 0.34, *p* = 0.56, ηp^2^ = 0.01)	(*F* = 0.90, *p* = 0.35, ηp^2^ = 0.02)	(*F* = 1.74, *p* = 0.19, ηp^2^ = 0.04)

a: significant difference from pre to post at *p* < 0.05; b: significant difference between the two groups at p < 0.05. CoV, coefficient of variation; CP, concentration performance; E, Total number of errors; EEG-MWI, EEG mental workload index; HF, high-frequency power; KSS, Karolinska Sleepiness Scale; LF, Low-Frequency power; LF/HF ratio, Ratio of Low Frequency to High Frequency; Mean HR, Mean Heart Rate; Mean RR, Mean RR interval; N- Lapses, Number of lapses; OPT-RT, Optimum response times; PVT, Psychomotor Vigilance Task; RMSSD, Root mean square of successive differences; RRT, reciprocal response time; RT, reaction time; SDNN, standard deviation of NN intervals.

### 3.4 Mid-term effects of using Aeris^®^-meeting-environment

#### 3.4.1 Resting EEG

The mid-term effect of using Aeris^®^-meeting-environment on resting brain activity are presented in Table 4 for theta, alpha, beta and gamma frequencies, respectively. Significant main effects of “groups” were found for beta frequency at the Fronto-C region (*p* = 0.04, np2 = 0.10) and for gamma frequency at the Frontal (*p* = 0.04, np2 = 0.11) and Fronto-C (*p* = 0.01, np2 = 0.16) regions with no significant effects of “intervention” or “group” × “intervention” interaction (*p* > 0.05). This main effect indicates higher activation in beta and gamma frequency bands in CG at both baseline and post-intervention test sessions. However, as indicated by the *post hoc* analysis, these are slight differences that are not significant. There was no significant main effect of “group,” “intervention” or interaction “group” × “intervention” in theta- and alpha frequencies band (*p* > 0.05).

**TABLE 4:** Mid-term effect of using Aeris^®^-meeting-environment on EEG signal (μV) for theta, alpha, beta, and gamma frequencies recorded at the frontal, fronto-central, temporal, and occipito-parietal regions.

	Pre-Intervention		Post-Intervention		2 ways Anova		
	Control group	Dynamic group	Control group	Dynamic group	Interaction Group × Intervention (F, *p*-value, η p^2^)	Group effect (F, *p*-value, η p^2^)	Intervention effect (F, *p*-value, η p^2^)
**Theta frequency (N ± SD)**							
Frontal region	4.60 ± 1.21	4.75 ± 2.33	5.75 ± 1.78	5.29 ± 1.97	(*F* = 0.31, *p* = 0.58, ηp^2^ = 0.007)	(*F* = 0.08, *p* = 0.78, ηp^2^ = 0.002)	(*F* = 2.34, *p* = 0.14, ηp^2^ = 0.06)
Fronto-central region	4.06 ± 1.25	4.55 ± 1.41	4.47 ± 2.88	4.85 ± 2.02	(*F* = 1.26, *p* = 0.27, ηp^2^ = 0.03)	(*F* = 0.005, *p* = 0.94, ηp^2^ = 0.0001)	(*F* = 3.05, *p* = 0.09, ηp^2^ = 0.07)
Temporal region	1.78 ± 2.43	2.82 ± 1.39	3.24 ± 1.24	3.30 ± 1.25	(*F* = 0.92, *p* = 0.34, ηp^2^ = 0.02)	(*F* = 1.15, *p* = 0.29, ηp^2^ = 0.03)	(*F* = 3.59, *p* = 0.07, ηp^2^ = 0.08)
Parieto-occipital region	3.10 ± 2.46	3.35 ± 1.39	4.07 ± 1.97	4.14 ± 1.35	(*F* = 0.03, *p* = 0.88, ηp^2^ = 0.0006)	(*F* = 0.08, *p* = 0.78, ηp^2^ = 0.002)	(*F* = 2.39, *p* = 0.13, ηp^2^ = 0.06)
**Alpha frequency**							
Frontal region	5.32 ± 2.62	3.90 ± 2.47	6.07 ± 1.94	4.80 ± 2.99	(*F* = 0.01, *p* = 0.92, ηp^2^ = 0.0002)	(*F* = 3.13, *p* = 0.08, ηp^2^ = 0.07)	(*F* = 1.20, *p* = 0.28, ηp^2^ = 0.03)
Fronto-central region	4.86 ± 2.53	4.36 ± 1.86	5.70 ± 1.68	4.15 ± 2.73	(*F* = 0.59, *p* = 0.45, ηp^2^ = 0.02)	(*F* = 2.29, *p* = 0.14, ηp^2^ = 0.05)	(*F* = 0.22, *p* = 0.64, ηp^2^ = 0.005)
Temporal region	2.52 ± 3.00	2.56 ± 1.93	3.39 ± 1.29	2.70 ± 2.29	(*F* = 0.29, *p* = 0.59, ηp^2^ = 0.01)	(*F* = 0.23, *p* = 0.63, ηp^2^ = 0.01)	(*F* = 0.55, *p* = 0.46, ηp^2^ = 0.01)
Parieto-occipital region	5.66 ± 3.56	4.85 ± 2.92	6.31 ± 2.48	5.41 ± 3.33	(*F* = 0.003, *p* = 0.96, ηp^2^ = 0.0001)	(*F* = 0.84, *p* = 0.37, ηp^2^ = 0.02)	(*F* = 0.41, *p* = 0.52, ηp^2^ = 0.01)
**Beta frequency**							
Frontal region	0.01 ± 1.61	−0.48 ± 2.40	0.54 ± 1.2	−0.88 ± 2.26	(*F* = 0.65, *p* = 0.43, ηp^2^ = 0.02)	(*F* = 2,80, *p* = 0.10, ηp^2^ = 0.07)	(*F* = 0.01, *p* = 0.91, ηp^2^ = 0.0003)
Fronto-central region	0.22 ± 1.29	−0.27 ± 1.45	0.86 ± 1.09	−0.74 ± 2.48	(*F* = 1.27, *p* = 0.27, ηp^2^ = 0.03)	(*F* = 4.54, *p* = 0.04, ηp^2^ = 0.10)	(*F* = 0.03, *p* = 0.87, ηp^2^ = 0.001)
Temporal region	−1.91 ± 3.49	−1.66 ± 1.66	−1.22 ± 1.35	−1.74 ± 1.90	(*F* = 0.31, *p* = 0.58, ηp^2^ = 0.01)	(*F* = 0.04, *p* = 0.85, ηp^2^ = 0.001)	(*F* = 0.19, *p* = 0.66, ηp^2^ = 0.01)
Parieto-occipital region	−0.92 ± 2.86	−1.13 ± 2.09	−0.84 ± 1.41	−0.69 ± 1.85	(*F* = 0.08, *p* = 0.78, ηp^2^ = 0.002)	(*F* = 0.003, *p* = 0.96, ηp^2^ = 0.0001)	(*F* = 0.17, *p* = 0.68, ηp^2^ = 0.004)
**Gamma frequency**							
Frontal region	−1.57 ± 1.73	−2.82 ± 2.30	−1.52 ± 0.98	−2.72 ± 2.33	(*F* = 0.002, *p* = 0.97, ηp^2^ = 0.0001)	(*F* = 4.70, *p* = 0.04, ηp^2^ = 0.11)	(*F* = 0.02, *p* = 0.89, ηp^2^ = 0.001)
Fronto-central region	−0.99 ± 1.53	−2.18 ± 1.48	−0.79 ± 1,12	−2.52 ± 2.73	(*F* = 0.25, *p* = 0.62, ηp^2^ = 0.01)	(*F* = 7.37, *p* = 0.01, ηp^2^ = 0.16)	(*F* = 0.02, *p* = 0.89, ηp^2^ = 0.001)
Temporal region	−2.85 ± 3.84	−3.42 ± 1.36	−2.47 ± 1.44	−3.27 ± 2.27	(*F* = 0.03, *p* = 0.88, ηp^2^ = 0.001)	(*F* = 0.84, *p* = 0.37, ηp^2^ = 0.02)	(*F* = 0.13, *p* = 0.72, ηp^2^ = 0.003)
Parieto-occipital region	−2.56 ± 3.01	−3.50 ± 1.77	−2.81 ± 1.52	−3.24 ± 2,31	(*F* = 0.15, *p* = 0.71, ηp^2^ = 0.004)	(*F* = 1.02, *p* = 0.32, ηp^2^ = 0.03)	(*F* = 0.0001, *p* = 0.99, ηp^2^ < 0.001)

**FIGURE 6:**
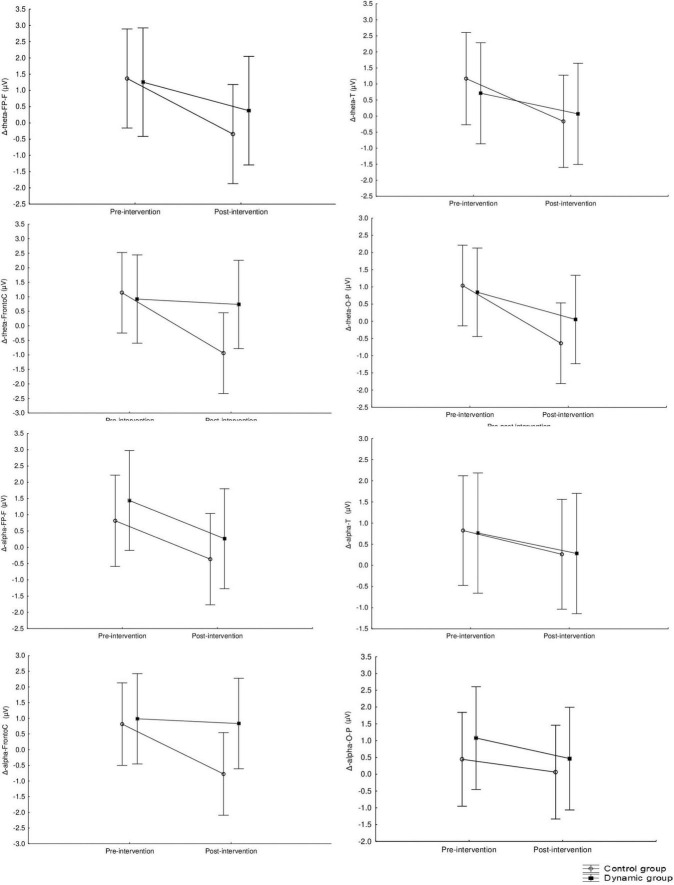
Mid-term effect of using Aeris^®^-meeting-environment on Δ (pre-post intervention) on theta and alpha frequency recorded at the frontal, fronto-central, temporal and parieto-occipital regions.

**FIGURE 7:**
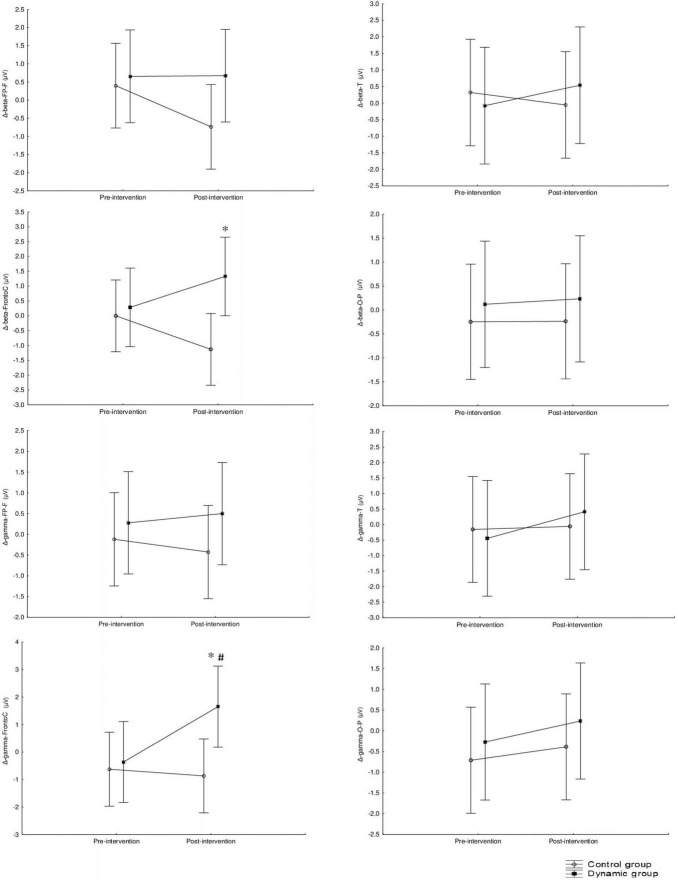
Mid-term effect of using Aeris^®^-meeting-environment on Δ (pre-post intervention) on beta and gamma frequency recorded at the frontal, central, temporal and occipital-parietal regions. *Significant difference between CG and DG at *p* < 0.05. ^#^Significant difference between pre and post intervention at *p* < 0.05.

The mid-term effects of using Aeris^®^-meeting-environment on Δ (pre-post 90 min) brain activation at resting state are presented in Figure 6 for theta and alpha and Figure 7 for beta and gamma frequencies. Significant main effects of “groups” were found for beta (*F* = 4.78, *p* = 0.03, np2 = 0.11) and gamma (*F* = 4.0, *p* = 0.05, np2 = 0.10) frequency bands at the Fronto-C region with no significant effect of “intervention” or “group” × “intervention” interaction. This main effect indicates higher Δ activation during the post-intervention session in beta and gamma frequency bands at the Fronto-C region. *Post hoc* analysis showed a statistically significant difference between CG and DG during the post-intervention session in Δ activation in beta (*z* = −2.41, *p* = 0.016, *d* = 1.10) and gamma (*z* = −2.34, *p* = 0.019, *d* = 0.94) frequency bands at the Fronto-C region with higher change in DG. Additionally, a significant difference between pre- and post- intervention was computed for Δ activation in beta frequency bands at the Fronto-C region of the DG (*z* = −2.09, *p* = 0.04, *d* = 1.08). There were no significant main effects of “group,” “intervention” or interaction “group” × “intervention” in theta and alpha frequencies bands (*p* > 0.05).

**FIGURE 8:**
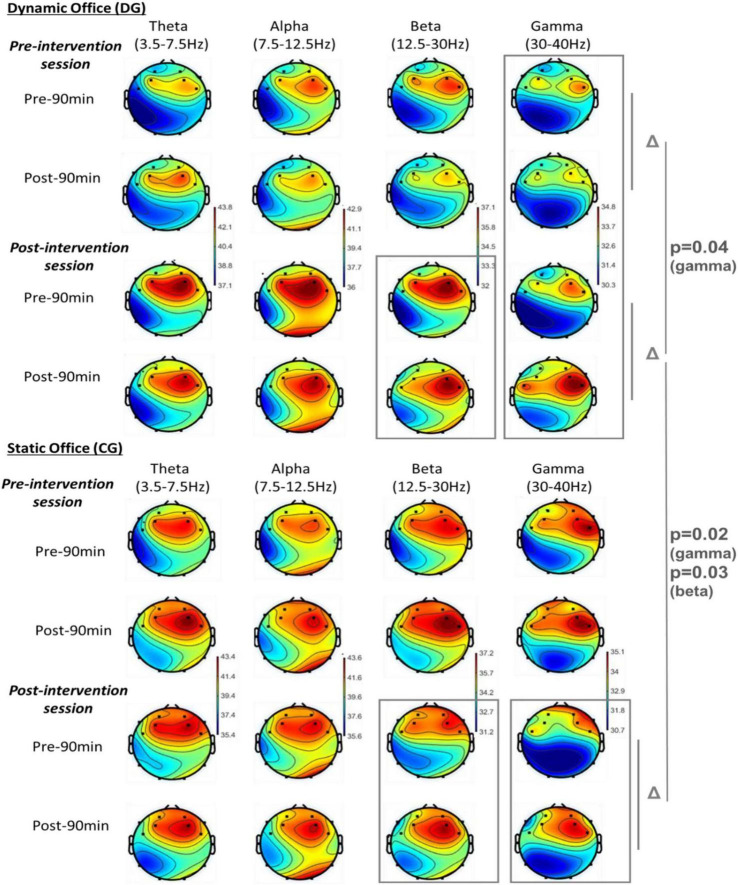
EEG brain activity for the theta, alpha, beta, and gamma bands in the dynamic and static offices at resting state.

Figure 8 summarizes the above-mentioned results and represents a mapping of the EEG brain activity for the theta, alpha, beta, and gamma bands in the dynamic and static offices at resting state during the pre- and post-intervention test sessions (at both pre- and post-90 min meetings).

#### 3.4.2 Cognitive performances

##### 3.4.2.1 Attentional performance (d2)

The mid-term effects of using Aeris^®^-meeting-environment on concentration performance (CP) and total number of errors (E) are presented in Table 5.

**TABLE 5:** The mid-term effect of using Aeris^®^-meeting-environment on d2 attentional performance, PVT vigilance performance, HRV’s time and frequency domain parameters and CoV, and sleepiness (KSS) and mental workload (EEG-MWI).

		Pre-Intervention		Post-Intervention		2 ways Anova		
		Control group	Dynamic group	Control group	Dynamic group	Interaction Group × Intervention (F, *p*-value, η p^2^)	Group effect (F, *p*-value, η p^2^)	Intervention effect (F, *p*-value, η p^2^)
**Cognitive performance**								
D2	CP	156.2 ± 21.38	169.64 ± 34.43	197.5 ± 28.28a	198.82 ± 35.33a	(*F* = 0.41, *p* = 0.53, ηp^2^ = 0.01)	(*F* = 0.61, *p* = 0.44, ηp^2^ = 0.02)	(*F* = 13.87, *p* = 0.001, ηp^2^ = 0.27)
	E	20 ± 9.71	23.5 ± 14.48	11.4 ± 5.50	20.6 ± 14.45	(*F* = 0.71, *p* = 0.41, ηp^2^ = 0.02)	(*F* = 2.77, *p* = 0,10, ηp^2^ = 0,07)	(*F* = 2.29, *p* = 0.14, ηp^2^ = 0.06)
PVT	RT (ms)	360.73 ± 29.54	343.96 ± 25.00	367.64 ± 28.56	353.36 ± 34.79	(*F* = 0.02, *p* = 0.89, ηp^2^ = 0.001)	(*F* = 3.06, *p* = 0.09, ηp^2^ = 0.07)	(*F* = 0.85, *p* = 0.36, ηp^2^ = 0.02)
	RRT (sec^–1^)	2.78 ± 0.21	2.77 ± 0.17	2.77 ± 0.20	2.85 ± 0.27	(*F* = 0.10, *p* = 0.75, ηp^2^ = 0.003)	(*F* = 2.45, *p* = 0.13, ηp^2^ = 0.06)	(*F* = 0.35, *p* = 0.56, ηp^2^ = 0.01)
	OPT-RT (ms)	303.18 ± 29.64	298.09 ± 23.72	296.77 ± 25.99	300.89 ± 32.12	(*F* = 0.30, *p* = 0.60, ηp^2^ = 0.01)	(*F* = 0.003, *p* = 0.96, ηp^2^ = 0.0001)	(*F* = 0.05, *p* = 0.83, ηp^2^ = 0.001)
	N-Lapses	1.36 ± 1.29	2.82 ± 2.96	2.91 ± 2.77	2 ± 1.95	(*F* = 2.81, *p* = 0.10, ηp^2^ = 0.07)	(*F* = 0.15, *p* = 0.70, ηp^2^ = 0.004)	(*F* = 0.27, *p* = 0.61, ηp^2^ = 0.01)
**HRV’s time domain**								
Mean HR (bpm)		76.23 ± 14.43	69.68 ± 6.82	76.19 ± 10.14	72.42 ± 12.54	(*F* = 0.17, *p* = 0.69, ηp^2^ = 0.004)	(*F* = 2.28, *p* = 0.14, ηp^2^ = 0.05)	(*F* = 0.16, *p* = 0.70, ηp^2^ = 0.004)
Mean RR (ms)		809.27 ± 132.90	868.83 ± 87.35	801.97 ± 121.52	846.64 ± 118.15	(*F* = 0.05, *p* = 0.83, ηp^2^ = 0.001)	(*F* = 2.21 *p* = 0.15, ηp^2^ = 0.05)	(*F* = 0.18, *p* = 0.68, ηp^2^ = 0.004)
SDNN (ms)		39.42 ± 11.33	45.56 ± 16.09	38.63 ± 13.63	41.40 ± 17.59	(*F* = 0.14, *p* = 0.71, ηp^2^ = 0.004)	(*F* = 0.99, *p* = 0.33, ηp^2^ = 0.02)	(*F* = 0.31, *p* = 0.58, ηp^2^ = 0.01)
RMSSD (ms)		37.15 ± 11.33	45.78 ± 18.57	37.29 ± 16.72	39.20 ± 18.57	(*F* = 0.45, *p* = 0.51, ηp^2^ = 0.01)	(*F* = 1.11, *p* = 0.30, ηp^2^ = 0.03)	(*F* = 0.42, *p* = 0.52, ηp^2^ = 0.01)
**HRV’s Frequency domains and covariance**								
LF (ms^2^)		49.96 ± 17.62	46.77 ± 19.83	54.43 ± 21.66	53 ± 18.35	(*F* = 0.02, *p* = 0.88, ηp^2^ = 0.001)	(*F* = 0.16, *p* = 0.70, ηp^2^ = 0.004)	(*F* = 0.84, *p* = 0.37, ηp^2^ = 0.02)
HF (ms^2^)		50.03 ± 17.61	53.19 ± 19.83	45.56 ± 21.66	46.98 ± 18.35	(*F* = 0.02, *p* = 0.88, ηp^2^ = 0.001)	(*F* = 0.15, *p* = 0.70, ηp^2^ = 0.003)	(*F* = 0.83, *p* = 0.37, ηp^2^ = 0.02)
LF/HF ratio		1.27 ± 0.88	2.04 ± 4.08	2.02 ± 2.50	2.41 ± 4.41	(*F* = 0.04, *p* = 0.85, ηp^2^ = 0.001)	(*F* = 0.35, *p* = 0.56, ηp^2^ = 0.01)	(*F* = 0.32, *p* = 0.58, ηp^2^ = 0.01)
CoV (%)		0.05 ± 0.01	0.05 ± 0.02	0.05 ± 0.02	0.05 ± 0.02	(*F* = 0.52, *p* = 0.47, ηp^2^ = 0.01)	(*F* = 0.03, *p* = 0.86, ηp^2^ = 0.001)	(*F* = 0.29, *p* = 0.59, ηp^2^ = 0.01)
**Sleepiness and mental workload**								
KSS		4.75 ± 2.45	4.36 ± 1.80	4.75 ± 2.30	5.27 ± 1.62	(*F* = 0.0000, *p* = 1.0000, ηp^2^ < 0.001)	(*F* = 0.38, *p* = 0.54, ηp^2^ = 0.01)	(*F* = 0.0000, *p* = 1.0000, ηp^2^ < 0.001)
EEG-MWI		0.65 ± 1.72	2.90 ± 2.56b	1.61 ± 1.07	2.55 ± 2.54	(*F* = 1.15, *p* = 0.29, ηp^2^ = 0.03)	(*F* = 6.86, *p* = 0.01, ηp^2^ = 0.15)	(*F* = 0.25, *p* = 0.62, ηp^2^ = 0.01)

a: significant difference from pre to post at p < 0.05; b: significant difference between the two groups at p < 0.05. CoV, coefficient of variation; CP, concentration performance; E, Total number of errors; EEG-MWI, EEG mental workload index; HF, high-frequency power; KSS, Karolinska Sleepiness Scale; LF, low-frequency power; LF/HF ratio, Ratio of Low Frequency to High Frequency; Mean HR, Mean Heart Rate; Mean RR, mean RR interval; N- Lapses, Number of lapses; OPT-RT, Optimum response times; PVT, Psychomotor Vigilance Task; RMSSD, root mean square of successive differences; RRT, reciprocal response time; RT, reaction time; SDNN, standard deviation of NN intervals.

A significant main effect of “intervention” was only found in CP (*p* = 0.001, np2 = 0.27) with no significant effect of “group” or “group × intervention” interaction. This main effect reflects significant increases in CP following the 2-week intervention periods in both CG and DG groups with *p* < 0.03 as indicated by the *post hoc* results. However, there were no significant differences between groups at any testing points. There were no significant main effects of “group,” “meeting-session” or interaction “group” × “intervention” in the total number of errors (*p* > 0.05).

##### 3.4.2.2 PVT

The mid-term effect of using Aeris^®^-meeting-environment on PVT vigilance performance are presented in Table 5.

There were no significant main effects of “group,” “intervention” or interaction “group” × “intervention” in any of the tested parameters (RT, RRT, OPT-RT, and N-Lapses) (*p* > 0.05). From pre- to post- intervention, a slight decrease in N-lapses was observed in DG, while CG showed a slight increase. However, these changes were not significant, as indicated in the *post hoc* results (*p* > 0.05). Similarly, no significant differences were shown between groups in any of the testing points (*p* > 0.05).

#### 3.4.3 Heart rate variability parameters (time and frequency domains)

##### 3.4.3.1 Time domains

The mid-term effect of using Aeris^®^-meeting-environment on HRV’s time and frequencies domain parameters are presented in Table 5.

ANOVA analysis showed no significant main effect of “intervention” and “group” as well as no “group” × “intervention” interaction for all tested parameters. *Post hoc* analysis showed no significant changes from pre- to post- intervention as well as no significant differences between groups at any of the measurement points (*p* > 0.05). Similarly, no significant difference was found between groups from pre- to post-intervention.

#### 3.4.4 Sleepiness and mental workload

The mid-term effect of using Aeris^®^-meeting-environment on KSS and EEG-MWI are presented in Table 4. A significant main effect of “groups” was only found for EEG-MWI (*p* = 0.01, np2 = 0.15) with no significant effect of “intervention” or “group × intervention” interaction. *Post hoc* analysis indicated a significant difference between CG and DG at pre-intervention with higher EEG-MWI value for DG (*p* = 0.01, *d* = 1.05). This difference was blinded after the 2 weeks intervention as a result of a non-significant slight increase in CG and a non-significant slight decrease in DG. There was no significant main effect of “group,” “intervention” or interaction “group × intervention” for KSS, as well as for Δ KSS and Δ EEG-MWI from pre- to post-intervention.

#### 3.4.5 Local experienced discomfort

**FIGURE 9:**
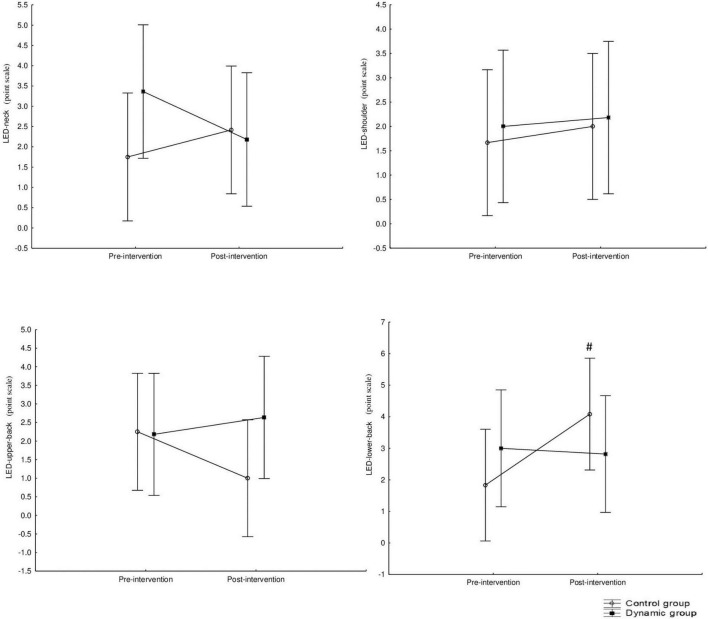
The mid-term effect of using Aeris^®^-meeting-environment on local discomfort. ^#^Significant difference between pre- and post-intervention. LED, local experienced discomfort.

The mid-term effect of using Aeris^®^-meeting-environment on local experienced discomfort are presented in Figure 9.

There were no significant main effects of “group,” “intervention” or interaction “group” × “intervention” in all LED parameters (i.e., discomfort in the neck, shoulder, upper or lower back) (*p* > 0.05). *Post hoc* analysis indicated a significant increase from pre- to post- intervention only in LED-lower-back in CG (*p* = 0.05, *d* = 0.78) but not in DG.

## 4 Discussion

The purpose of the present study was to assess the acute and mid-term effects of the Aeris^®^-meeting- environment, a standing-based configuration of a height-adjustable meeting desk (Aeris^®^ meeting desk), active floor mats (Aeris^®^ mEEGmat), and ergonomic Aeris^®^ muvmat chairs, on brain activity, cognitive performance, HRV, sleepiness, mental workload, and local experienced discomfort in healthy adults.

The main results revealed that, compared to the CG, the DG showed higher Δ (pre-post 90 min-meeting) in fronto-central beta and gamma frequencies at post-intervention. The difference in gamma frequency was accompanied by a significant increase in Δ (pre-post 90 min-meeting) from pre- to post-intervention only in the DG group. These results indicate that 2 weeks of using the dynamic meeting environment from Aeris^®^ has the potential to increase beta and gamma bands, particularly in the fronto-central region, during the 90 min meeting session. The current increase in this specific region suggests an increase in the subject’s willingness to concentrate and to deal with a higher flow of information (Henz and Schöllhorn, 2019). Indeed, previous studies have demonstrated the important role of the frontal lobe in many higher-level functions of the brain, including executive functions, voluntary control, and action planning, as well as speech and language production, working memory, reasoning and judgment, organization and planning, problem-solving, controlling social behaviors, etc. (Jung et al., 2000; Cardoso de Oliveira, 2002; Moriguchi and Hiraki, 2013). Given that most of these functions are the basis of any meeting session and that the frontal lobe is responsible for maintaining social appropriateness (El-Baba and Schury, 2023), it’s understandable that the main significant effects of the dynamic office on brain activation occurred in the fronto-central region, particularly since the participants worked with partners throughout the whole intervention period. It should also be mentioned that this region is currently tested based on data collected from the FC5 and FC6 electrodes, where the primary motor cortex (M1), relevant for high cognitive processes (Bhattacharjee et al., 2021), is located.

The present mid-term effects of the dynamic office environment were mainly observed at the beta and gamma frequencies. According to previous studies, beta activity is associated with an increased readiness of the cognitive system for attentional performance, especially in short- and long-term concentration tests (Wróbel et al., 2007; Sauseng and Klimesch, 2008; Saleh et al., 2010). Gamma activity also occurs during concentration testing as a correlate for selective attention (Engel and Singer, 2001; Varela et al., 2001), which is an indicator of strong concentration and a high flow of information. It has also been shown that increased EEG beta oscillation plays a crucial role in enhancing feedback loops of visual information processing at subsequent stages (Gola et al., 2013), while increased gamma activity enhances selective attentional processing (Engel and Singer, 2001; Varela et al., 2001).

Together, we thought that increasing beta and gamma activities in a dynamic meeting environment would make the DG better at paying attention and staying alert and vigilant than the CG. Our hypothesis was partially supported, and the present findings revealed that the use of the dynamic office affects the vigilance performance (i.e., maintenance effect) more than the attentional one. Indeed, the use of the Aeris^®^-meeting-environment during the 90 min meeting session seems to maintain PVT vigilance performance, as evidenced by a significant increase in N-lapses from pre- to post- 90 meetings, only in the CG (2.5 more laps on average), which was not the case in the DG. Furthermore, following the 2-week interventions, a slight decrease in N-lapses was observed in DG, while CG showed a slight increase. However, these changes were not significant. Although a main effect of “90 min meeting” and “intervention” was found in concentration performance measured using the attentional test “d2,” indicated increased performance from pre- to post-90 min meeting (i.e., acute effect) as well as from pre- to post-intervention (i.e., mid-term effect) in both tested groups, there were no differences between CG and DG at any of the test points.

Previous studies have consistently shown positive correlations between engagement in leisure and/or social activities and cognitive performance. Research involving middle-aged adults has demonstrated that participating in leisure activities that involve mental engagement, such as brain teasers, courses, chess, or bridge, as well as engaging in social activities like volunteer work, can lead to improved performance on various cognitive tests related to executive function (Richards et al., 2003; Singh-Manoux et al., 2003; Small et al., 2006). In the context of the current study, the employed 90-min meeting protocol incorporates both leisurely and socially oriented problem-solving tasks, such as collaborative games. This setting is expected to foster creativity, interactions, and collaboration among participants, and thereby contribute to the observed acute and mid-term cognitive enhancements in both groups (Kelly et al., 2017; Evans et al., 2019; Zhaoyang et al., 2021).

On the other hand, the slight, non-significant advantage of DG over CG in terms of enhanced cognitive performance may be attributed to the reduction in sitting times facilitated by the dynamic environment. Indeed, research has indicated that prolonged and excessive sitting at workplaces can lead to vascular and cardiometabolic changes that predispose to both peripheral and central vascular inflammation and poor cortical perfusion, which in turn can be associated with decreased cognitive function (Chandrasekaran et al., 2021). Conversely, interrupting extended sitting periods with short bouts of movement is suggested to regulate peripheral and cerebral blood flow, enhance endothelial functions and improve venous return (Carter et al., 2018; Paterson et al., 2020). This is viewed as a preventive strategy to counteract impaired cognitive functions linked to prolonged sitting (Carter et al., 2018). In a recent meta-analysis by Paterson et al. (2020), the effects of interrupting prolonged sitting periods with various activities such as aerobics, resistance exercises, or standing on the flow-mediated dilation of brachial, femoral, and posterior tibial arteries were examined. The meta-analysis revealed a significant improvement in flow-mediated dilation during interrupted sitting bouts compared to uninterrupted sitting. However, the precise dose-response relationship between interventions aimed at reducing sitting time and enhancing physical activity and their impact on cerebral flow velocity remains unclear. These findings suggest that the interruption movement protocol of sitting bouts used in the present intervention may not have been optimized to yield a significant difference in cognitive response between the DG and the CG, as only a slight advantage in favor of the DG was observed. This highlights the need for further research to refine the sitting interruption protocol that yields the most pronounced cognitive benefits in dynamic office settings.

The present results partially support previous neurophysiological findings by our department on the beneficial effects of a dynamic office environment (Aeris Active Office, consisting of a sitting and a standing desk that are height adjustable) on cognitive functions and brain oscillations (Henz and Schöllhorn, 2019). In that study, the EEG data showed an increase in theta, alpha, gamma, and/or beta activities in the dynamic office, which was accompanied by increased attentional performance after 120 min of working as well as increased attentional and vigilance performance after the 2-week intervention (Henz and Schöllhorn, 2019).

In that study, we interpreted the increases in theta, alpha, and/or beta oscillations during the cognitive tasks (attentional and/or vigilance) as signs of changes in visual attention caused by more motor activity when working in a dynamic office that encourages physical activity. In the same way, we interpreted the improvement in cognitive functions as the result of an enhancing effect of motor activity on attentional processing using the dynamic office. Particularly, it was mentioned that, in contrast to a static office environment, performing attentional tasks in a dynamic office environment stimulates executive cognitive controlled processing as a result of the activation of more areas in the brain through increased movement and sensory variety as well as stimulation by the Active Floors (MuvMat).

The findings of the present study support the beneficial effect of using a dynamic office environment on beta and gamma activities following attentional and vigilance cognitive tasks but don’t support similar effects on alpha and theta activities. Similarly, the present findings partially support the beneficial effects of such an environment on vigilance performance, particularly its maintenance effect, but don’t support a similar effect on attentional performance. The discrepancies between findings regarding alpha and theta activities as well as attentional performance can be explained by the limited physical activity in the current dynamic environment compared to the one in Henz and Schöllhorn (2019) study. Indeed, to be closer to the real meeting environment, the participants in the present study were free to either practice some movement on the MuvMat or just switch between the standing and high-sitting positions, but there was no strict or guided instruction to move during the meeting session. Therefore, it can be suggested in future research to test whether increased movement instructions in the same environment would enhance beta, gamma, as well as theta and alpha activities in DG compared to CG and whether such enhancement would result in higher attentional and vigilance performances. Additionally, to what extent these differences were due to individual characteristics of the sample or to a higher frequency of position changes requires further research. The more frequent changes seem to stimulate the lower frequencies more strongly, which are responsible for the integration of different areas, among other things. It would also be of interest in future studies to investigate to what extent the different tasks, and specifically the games and social interactions, led to increased and altered activation of the medial regions of the prefrontal cortex, specifically related to social behavior. Maybe it is about the relative amount of movement and social stimuli.

It is well known that theta activity is related to behaviors that demand action planning based on received sensory information (Liepert et al., 1998; Caplan et al., 2003). Chung et al. (2017) talked about increased theta activity in the frontal parts of the brain as a neural basis for improvements in cognitive control, like paying attention and preparing to execute movement. In particular, frontal theta activity shows up in the early allocation of selective attention resources to external visual stimuli. This has been shown to improve cognitive control during visuo-motor tasks (Berchicci et al., 2015) and goal-directed attention (Dowdall et al., 2012). On the other hand, several studies have shown that alpha activity in parietal regions is involved in the processing of visual stimuli (Jensen et al., 2012; Roux and Uhlhaas, 2014; Foster et al., 2016). Further, there is evidence for a relationship between alpha oscillations and voluntary attentional allocation (Worden et al., 2000; Kelly et al., 2006; Thut et al., 2006; Foxe and Snyder, 2011).

Therefore, it would be interesting, during the meeting session, to create stimulus-induced changes in the beta and gamma bands as well as in the theta and alpha bands in order to correlate for modulation of goal-directed spatial attention (Harris et al., 2017). This would be possible, as mentioned above, by combining the current Aeris dynamic meeting environment with additional motor movements on MuvMat. According to the system dynamic approach, which considers deviations as constructive fluctuations, it’s preferable that such movements change continuously during task performance and problem solving in order to increase fluctuations and deviations, which may stimulate transitions in brain state and thereby enhance cognitive performance (Schöllhorn, 2000). This assumption is in line with recent neurophysiological studies showing that differential movement training inducing increased fluctuations in the human body increases brain activity in the theta and alpha ranges as well as learning rates (Henz and Schöllhorn, 2016; Henz et al., 2018). However, further studies are needed to test this hypothesis. Additionally, besides the resting state analysis (i.e., the current report), further analysis of the brain activity during each cognitive test (attentional and vigilance) is warranted.

Importantly, the present findings showed that the acute use of the Aeris^®^-meeting environment during the 90-min meeting session was accompanied by a lower sleepiness score compared to the conventional environment. These results are in line with those of Henz and Schöllhorn (2019), who reported a decrease in wakefulness during the post-test compared to the pre-test when working in a static office environment. Taking into consideration that this wakefulness effect occurred with increased brain activity, it can be assumed that working in an active or dynamic environment activates the brain in a larger area, and thus creative solutions are more likely to take place as tasks can be solved using multiple sensors and diverse resources, thereby making workflows appear less monotonous, which may reduce sleepiness and increase wakefulness. Furthermore, these findings suggest that sitting-reduction strategies such as substituting sitting with standing (e.g., Aeris Active Office) targeting increased standing, stepping/moving, or both, may benefit alertness and reduce sleepiness sensations during daily office work or meeting sessions. Previous studies showed that excessive sitting was associated with altered cerebral blood flow and cognitive function (Wheeler et al., 2017). By reducing prolonged sitting time, enhanced cerebral blood flow is expected, which may be the origin of enhanced alertness and reduced sleepiness. However, these speculations need to be evaluated in future neurophysiological studies. Additionally, it is well known that sleepiness occurs with tiredness and that moving is one of the best ways to beat tiredness (Rosenthal et al., 2008; Braley et al., 2012). It can therefore be assumed that meeting or working in a dynamic office may prevent mental tiredness and sleepiness by constantly engaging the person in new intellectual challenges while avoiding monotony.

In the same way, the mid-term use of the present dynamic meeting environment showed to blind the higher baseline values of EEG-MWI recorded in DG compared to CG, as a result of a non-significant slight increase of EEG-MWI in CG and a slight decrease in DG during the 2-week intervention period. These results indicate a less mentally exhausting meeting using a dynamic meeting environment and provide neurophysiological support for the finding of Henz and Schöllhorn (2019) showing greater subjectively perceived calm using a dynamic office. In addition to the few neurobehavioral-related positive aspects described above, encouraging results have also been found regarding the promotion of health aspects when meeting in the dynamic office. Indeed, the mid-term use of the Aeris^®^-meeting environment also showed to prevent lower-back discomfort following 2 weeks of consecutive meetings (5 meetings of 90 min/week), but with no significant effect on neck, shoulder, or upper-back discomfort.

The present findings are in line with previous ones from Renaud et al. (2020), showing that the sit-stand desk improved posture and reduced low back pain among office workers with chronic low back pain, as well as with the findings from Henz and Schöllhorn (2019), in healthy office workers, showing a possible relaxed and upright posture while working in the Active Office, as measured by the EMG activity. Both findings suggest the use of dynamic office environments in everyday work and/or meetings as promising seat-reduction strategies that may promote a healthier, stronger, and more stable spine. Indeed, prolonged sitting posture is well known to be highly risky for the skeleton, joints, and muscles, and therefore, it should be avoided. At the skeleton level, sitting for long periods of time challenges and eases the bones and thereby can lead to osteoporosis (Malinska et al., 2021). At the joint level, the longer a person sits, the more likely they are to develop arthritis (inflammatory joint disease) and osteoarthritis (joint deformity), as our joints are not designed for monotonic and repetitive movements (Malinska et al., 2021). Therefore, the most suggested antidote for osteoporosis is movement. At the muscular level, by holding a certain posture for a long time, muscle tissue becomes tight and absorbs more energy than healthy, flexible muscles (Jung et al., 2020). Additionally, weakened muscles, unable to withstand constant stress, were previously suggested as a leading cause and significant risk factor for low back pain in office workers (Teyhen et al., 2012; Cho et al., 2015; Dang et al., 2019).

Concerning the HRV parameters, the present findings revealed no significant acute or mid-term effects of using the Aeris^®^ meeting environment on HRV’s time and frequency domains (time domain: mean HR, mean RR, SDNN, and RMSSD; frequency domain: LF, HF, LF/HF, and COV). These results do not align with those of Henz and Schöllhorn (2019), showing a stronger activity in the HF range and a lower activity in the LF range of the HRV, using the dynamic office environment, indicating higher activity of the parasympathetic nervous system when working in such an environment, even on demanding tasks and tasks under time pressure. The limited beneficial effect of the dynamic meeting environment on HRV and mood dimensions, shown in the present report, further highlights the need for increasing movement in this environment and thereby the need for future research testing the effect of combining the current Aeris dynamic meeting environment with additional Muvmat motor movement on neurophysiological and behavioral responses and adaptation.

## 5 Strengths and limitations

This study represents a pioneering effort to assess both acute and mid-term effects of a dynamic meeting environment on cognitive performance while controlling neurophysiological responses, sleepiness, and discomfort during a simulated meeting session with social stimuli. However, it is essential to acknowledge several limitations that should be considered when interpreting the present findings. While confounding variables such as physical activity behaviors and sleep patterns were controlled both before and during the intervention, other covariates were not accounted for, including participants’ physical condition and dietary patterns. Additionally, the inter-group design, despite the g-power justification, could potentially impact the results due to the small sample size and the differences in the indicators. Furthermore, in the present study, the Polar H10 HR monitor and the wireless EEG headset Emotiv™ Epoc + were not synchronized, which means that time-synchronous measurements between both systems were not guaranteed. Another technical limitation of the current study can be linked to the use of the 14-channel Emotiv EEG system, given that EMOTIV electrode positions do not map the entire cortex. This has to be considered, especially when interpreting the brain maps in Figure 8. Since there are no electrodes in the central and parietal areas, the brain maps in these areas show interpolations of the surrounding electrodes. However, it should be noted that, in order to simultaneously record the three interlocutors, the EMOTIV EPOC + was the optimal, low-cost, validated solution that responded to the study design and objectives. Future studies in this field should take into account these aforementioned limitations to ensure more robust and less biased findings. Moreover, considering that the aim of the present study was to evaluate the overall impact of the dynamic meeting environment provided by Aeris GmbH rather than examining the impact of each component individually, future studies may explore the effect of each component separately. This could involve incorporating distinct experimental conditions, each involving one of the ergonomic instruments, such as the Aeris^®^-meeting-desk, Aeris^®^ muvmat, and Aeris^®^ muvman chairs, to gain a more comprehensive understanding of their individual contributions to cognitive performance and neurophysiological responses.

## 6 Conclusion and future direction

The use of the dynamic office environment from Aeris^®^ has the potential to increase beta and gamma bands in the fronto-central region, improve alertness, reduce mental exhaustion and low-back discomfort, and maintain vigilance performance during a simulated meeting session. These findings give preliminary support to the advantages of meeting in a dynamic meeting environment compared to a static meeting environment and support the assumption that working in a dynamic office activates the brain in a larger area without showing increased fatigue symptoms, so creative solutions during problem-solving tasks can take place with more diverse and less monotonous resources. However, in many other tested parameters, such as the theta and alpha bands, attentional performance, all HRV-related parameters, and discomfort in the neck, shoulder, and upper back, the acute as well as the mid-term use of the actual environment version seems to be ineffective. Therefore, it’s suggested, during the meeting session, to create stimulus-induced changes in a wide range of neurophysiological and behavioral parameters (e.g., alpha and theta bands, HRV, mood, etc.) in order to correlate for modulation of larger aspects of cognitive performance. This would be possible by combining the actual Aeris dynamic meeting environment with additional motor movement on MuvMat, such as differential motor movements inducing fluctuations in the human body, which was previously shown to increase brain oscillations in the theta and alpha ranges (Henz and Schöllhorn, 2016; Henz et al., 2018). However, further studies are warranted to test this hypothesis. The results of the present study as well as future studies in this field are of relevance in the field of neuroergonomics, for the design of office meeting environments that may help stimulate the brain toward an optimum psychophysiological level of activation and wakefulness necessary to optimize cognitive performance and achieve meeting goals while promoting healthy meetings.

## Data availability statement

The raw data supporting the conclusions of this article will be made available by the authors, without undue reservation.

## Ethics statement

The studies involving humans were approved by the local ethic committee at the Faculty of Human Science at Johannes-Gutenberg-University of Mainz. The studies were conducted in accordance with the local legislation and institutional requirements. The participants provided their written informed consent to participate in this study.

## Author contributions

AA: Conceptualization, Data curation, Formal analysis, Investigation, Methodology, Project administration, Resources, Software, Supervision, Validation, Visualization, Writing—original draft, Writing—review and editing. MS: Data curation, Formal analysis, Investigation, Software, Writing—review and editing. IF-F: Data curation, Formal analysis, Investigation, Software, Writing—review and editing. MB: Formal analysis, Software, Validation, Visualization, Writing—review and editing. KT: Conceptualization, Validation, Writing—review and editing. BB: Conceptualization, Formal analysis, Methodology, Software, Validation, Writing—review and editing. NR: Conceptualization, Data curation, Formal analysis, Investigation, Software, Visualization, Writing—review and editing. WS: Conceptualization, Investigation, Methodology, Project administration, Resources, Supervision, Validation, Visualization, Writing—review and editing.

## Footnotes

1
http://www.ipaq.ki.se

## Annex 1

### Content of the 90 min simulated meetings during the 2-week intervention period

The content of the intervention and test simulated meetings were based on differential team tasks including three or more tasks from different task categories. The main categories consist of:

(i) Discussion round: *Popular topics/an article/student related topics/Opinions (e.g., Job preferences)* □ *Argue pros and cons and vote for a team opinion*
(ii) Project based learning/Creative tasks/Brainstorming
  • *Design a study/sport lesson/treatment/marketing strategy*
  • *Discuss the current curricula modules and gave suggestions and recommendation*
  • *Present a topic as simple as possible (Eli5)*
  • Given a few words, create a short story containing them
  • Watch a muted video extracted from a film and try to recreate the conversation
(iii) Team building games
  • *LEGO serious play*
  • *Team health monitoring games*
  • *Marshmallow Spaghetti tower, Drawing games, Zoom, Classify, Rebuilt in Lego, etc.*
(iv) Cooperative games
  • Semantic word games (e.g., Connector)
  • Board/Card games (Escape room, The Game, Hanabi, etc.)
  • Mental representation of skilled action
  • Guessing the place of the ball/scored or failed
(v) Quizzes/Logic puzzles
  • Dog, Rice, Chicken/Where is the ball?/Who lives in the blue house?/Sport related
  • Hidden object/pattern/difference picture
